# Potential of Japanese Macaques for Understanding Etiology and Seasonality of Repetitive Linear Enamel Hypoplasia in Nonhuman Primates

**DOI:** 10.1002/ajp.23713

**Published:** 2024-12-17

**Authors:** Mark Fretson Skinner, Mao Asami, Matthew M. Skinner, Akiko Kato

**Affiliations:** ^1^ Archeology Simon Fraser University Burnaby British Columbia Canada; ^2^ Center for the Evolutionary Origins of Human Behavior Kyoto University (EHUB) Kyoto Japan; ^3^ Max Planck Institute for Evolutionary Anthropology Leipzig Germany; ^4^ Oral Anatomy, School of Dentistry Aichi Gakuin University Nagoya Aichi Japan

**Keywords:** dentition, developmental stress, *Macaca fuscata*, seasonality, Shimokita Peninsula

## Abstract

Japanese macaques are ideal to advance understanding of a wide‐spread pattern of recurrent developmental distress in great apes, preserved as repetitive linear enamel hypoplasia (rLEH). Not only are they numerous, unendangered, and well‐studied, but they are distributed from warm‐temperate evergreen habitats in southern Japan to cool‐temperate habitats in the north, where they are adapted behaviorally and phenotypically to winter cold and seasonal undernutrition. We provide a pilot study to determine if enamel hypoplasia exists in Japanese macaques from the north and, if temporal patterns of enamel hypoplasia are consistent with seasonal cold, undernutrition and/or exposure to secondary plant compounds. High‐resolution casts of canine teeth from 15 males obtained from Shimokita Peninsula (latitude 41.3° N) between 2012 and 2014, whose skeletons are curated at the Center for the Evolutionary Origins of Human Behavior, Kyoto University, were imaged by confocal and scanning electron microscopy. Perikymata, the surface expression of regularly deposited imbricational layers of enamel, provide an estimate of time between and within hypoplastic enamel defects. Based on histological sections from five individuals, we determined Retzius periodicity to be 7 days. Evidence for recurrence, duration, and severity of 68 LEH defects was collected from perikymata counts as well as measurements of LEH angle of onset, depth and width. Male canine teeth show four to five recurrent, evenly‐spaced enamel defects per crown with a median of 54.8 (range 18–74) perikymata between defects; lasting on average 8.7 (range 1–20) perikymata. These translate into repetitive developmental distress averaging every 1.05 years, lasting 8.7 weeks, less than local winter foraging conditions (100 days). We conclude that linear enamel hypoplasia recurs circ‐annually among high‐latitude male monkeys from Japan. The triad of cold, hunger and anti‐feedants can be differentiated in future study through recourse to provisioned and un‐provisioned populations throughout the Japanese archipelago.

## Summary

- Northern Japanese male macaque monkeys record circ‐annual developmental stress in dental enamel.
- Stress lasts about 2 months, thinning the affected enamel proportional to reported loss of body mass during winter dietary conditions.

## Graphical abstract

Circ‐annual enamel thinning in Japanese macaque canine tooth.

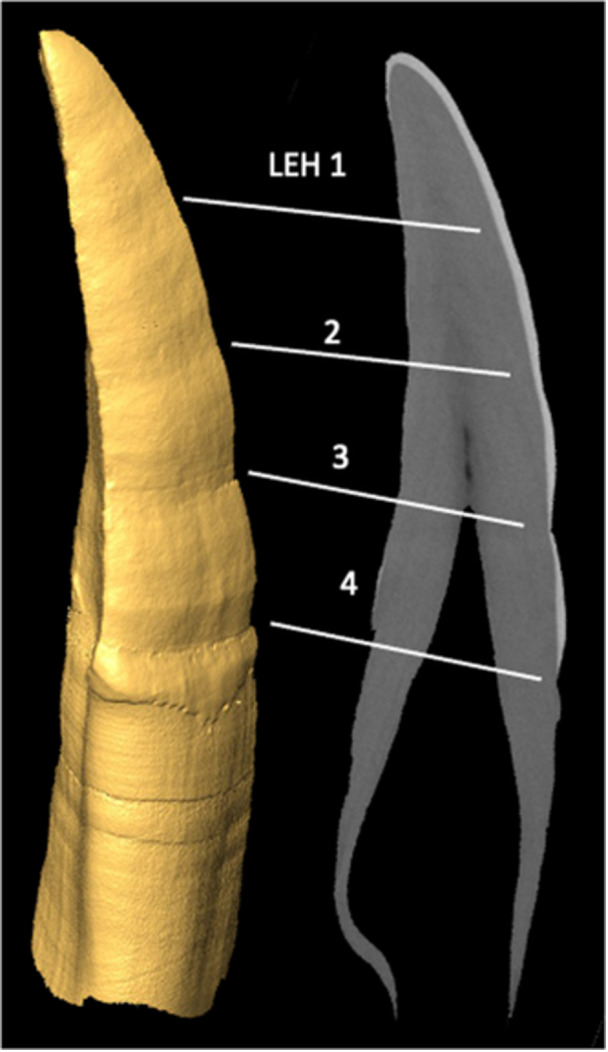

**Abbreviations**
CT: computed tomography
DSR: daily secretion rate
EDR: enamel deficit ratio
EHUB: Center for the Evolutionary Origins of Human Behavior, Kyoto University
PK: perikymata
rLEH: repetitive linear enamel hypoplasia
RP: Retzius periodicity
SEM: scanning electron microscope

## 1 Introduction

Enamel hypoplasia (EH) is a well‐documented malformation of enamel tissue that can manifest in a number of forms such as pitting, linear, and plane‐form defects (Boyde 1970; Hillson 2014; Lukacs 1999; Towle and Irish 2019). EH has been observed in numerous extant and fossil primate species and has been linked to a number of potential causes, including seasonal differences in nutrition (Brunet et al. 2002; Chollet and Teaford 2010), illness (Goodman, Martinez, and Chavez 1991; Skinner and Hopwood 2004), and cold‐stress (Skinner 2021). EH can be assessed at the population level by prevalence and, at the individual level, by timing as well as intensity. While previous studies of prevalence of linear EH (LEH) in nonhuman primates (Guatelli‐Steinberg and Skinner 2000; Hannibal and Guatelli‐Steinberg 2005; Skinner 1986) have not significantly advanced our understanding of etiology, the timing of recurrence and duration hold more promise, in that they can be more easily associated with documentable events. Repetitive linear EH (rLEH) is particularly promising and is commonly reported in both extant and fossil nonhuman primates (Guatelli‐Steinberg 2004; McGrath et al. 2015; O'Hara and Guatelli‐Steinberg 2020; Skinner and Skinner 2017); however, it continues to prove challenging to identify its underlying causal factor(s). In this study, we examine whether *Macaca fuscata* monkeys, a numerous and well‐documented species occupying a wide latitudinal, altitudinal and ecological range throughout the archipelago of Japan, could serve as a new model to help elucidate the etiology of rLEH.

The Japanese macaque is the most intensively studied non‐human primate in the world, including both wild and captive populations (Agetsuma and Nakagawa 1998; Aoki et al. 2015; Enari 2014; Enari and Sakamaki‐enari 2013; Fooden and Aimi 2005; Furuichi, Takasaki, and Sprague 1982; Hamada and Yamamoto 2010; Hanya 2010; Hanya et al. 2011; Kurihara et al. 2020; Nakagawa 1989; Nakagawa, Nakamichi, and Sugiura 2010; Tsuji 2010). Their relatively large numbers mean they are not an endangered species, although there are locally threatened populations noted in the Red Lists of the Ministry of the Environment, Japan (Sakamaki et al. 2011). They are distributed throughout most of Japan from latitude 30°–41° N at the northern tip of Honshu Island (Nakagawa, Nakamichi, and Sugiura 2010). Consequently, separate populations are adapted to a range of habitats from warm‐temperate evergreen forest to cool‐temperate deciduous forest in northeastern Japan (Hanya 2010), where they may have to cope with deep snow in the winter months (Yamagiwa 2010). Moreover, there are many provisioned primate colonies maintained throughout Japan in semi‐free ranging conditions on whom detailed, longitudinal studies of behavior, physiology, morphology and ontogeny are routinely performed (Aoki et al. 2015; Hamada and Yamamoto 2010; Nakayama et al. 1971; Nelson 2020; Sugiyama and Ohsawa 1982; Takeshita et al. 2018; Yamagiwa 2010). Lastly, Japanese macaque skeletons and teeth, drawn from these diverse climatic, ecological and nutritional habitats throughout Japan, exist in large numbers in the collections of the Center for The Evolutionary Origins of Human Behavior (EHUB), at Kyoto University.

### 1.1 Potential Causes of Developmental Stress in Japanese Macaques

There are comparatively few studies of enamel hypoplasia in monkeys (but see notable instances (Chollet and Teaford 2010; Guatelli‐Steinberg and Benderlioglu 2006; Macho et al. 1996; Newell 1998; O'Hara and Guatelli‐Steinberg 2020)). In a pioneering study of enamel hypoplasia in nonhuman primates, Newell (1998) found no enamel hypoplasia among one male and two female Japanese macaques. Only recently has a study of, specifically, plane‐form enamel defects among this species appeared (Towle et al. 2024). Of the three samples, only the Yakushima Island animals exhibit a remarkably high prevalence of plane‐form defects, which is attributed by the authors most likely to habitat disturbance. However, based on previous studies, we would suggest that consideration should also be given to possibly three inter‐acting, non‐anthropogenic stressors: winter cold, undernutrition, and malnutrition‐in the form of secondary plant compounds (SPCs).

## 2 Winter Cold

Japanese macaques experience a degree of thermo‐regulatory stress regardless of latitude. Even those macaques who live in the richest habitat available to the species, at Yakushima Island (30.3° N), show significant ketonuria in winter months, indicating that they are drawing on fat reserves in this season (MacIntosh et al. 2012). Fat reserves have a role in both insulation and nutrition. In the wild, cold and undernutrition usually co‐occur. Consequently, Japanese macaques show a wide range of phenotypic plasticity allowing them to adjust to local environmental challenges (Enari and Sakamaki‐Enari 2013). Macaques show a northward clinal gradient of decreasing tail length, longer, more dense and lighter pelage, with increasing body dimensions that correlate with lowest monthly average temperature. These characters likely reflect the selection for cold adaptation (Hamada and Yamamoto 2010). Furthermore, the timing of important developmental events in the Japanese macaque life cycle reflects phenotypic adaptation to cold conditions. They are strictly seasonal breeders, with most births occurring in May (but observed from February through August) (Hamada and Yamamoto 2010). Northern, new‐born individuals have markedly more dense hair (Fooden and Aimi 2005), which suggests a geographic genetic adaptation to the cold. There is evidence that Japanese macaques have long been adapted to marked seasonality and winter cold, having migrated half a million years ago from Korea (Hamada and Yamamoto 2010) and descended from an ancestral form with the cold‐adapted shorter tail of mainland relatives (Hamada and Yamamoto 2010). A lower canine tooth from the far northeast of the Shimokita Peninsula, referred to *M*. cf*. fuscata*, has been dated to the Late Pleistocene (ca. 120 K years ago) (Hamada and Yamamoto 2010).

While it seems Japanese macaques can adapt to winter cold, they are not considered to be specialized for solely cold areas, perhaps not having evolved in Japan sufficiently long to adapt to winter cold (Tsuji et al. 2015). Thus, they bear a high thermoregulatory cost in winter and adopt a risk‐averse strategy of reduced movement and enhanced reliance on bark and stored fat (Enari and Sakamaki‐enari 2013). Interestingly, Japanese macaques from Kamikochi and Shimokita in the north reduce travel time in the winter, choosing to expend less energy on seeking food and, rather, to concentrate on thermoregulation. The animals seem to be responding to reduced temperature, increased wind strength and deeper snow (Tsuji 2010). Further south, in Kyoto (35° N), a detailed study of temperature regulation among provisioned, but outdoor macaques, concluded that under less extreme winter conditions, Japanese macaques are remarkably efficient autonomic thermoregulators, just so long as they are not facing food shortages or extremely low temperatures (Sha et al. 2018).

Thermoregulation is critically important for the survival of macaques living in the northernmost habitats; for example, hot‐spring bathing has been adopted by some populations (Takeshita et al. 2018; Zhang, Watanabe, and Eishi 2007). Additionally, in some populations, sunbathing is preferred over huddling and foraging (Pflüger et al. 2019). More germane to our research, thermoregulation, among specifically juvenile Japanese monkeys, was studied in a cold‐adapted (and provisioned) population from Shiga Heights (800–1600 m), central Honshu Island (Hori et al. 1977). These juveniles showed no change in metabolic rate despite near‐freezing ambient temperatures and maintained a constant rectal temperature, which was attributable, not to innate characters, but to fur which grows thick and long from autumn to winter. It was concluded by these authors that the critical ambient temperature for a juvenile or adult Japanese macaque is below 0°C. A recent urinalysis study among semi‐free ranging, provisioned Japanese macaques from Inuyama (35.4° N), exposed to average outdoor winter temperature of 0.7°C and minimum of −3.7°C, showed significantly elevated levels of the freely circulating triiodothyronine, involved in autonomic temperature regulation (Thompson et al. 2017). Nelson (2020) recently confirmed that winter cortisol levels were ‘marginally higher’ in this same population, but not significantly so. Similarly, a troop of provisioned female Japanese macaques exposed to strong seasonal changes in temperature showed that the lower the temperature, the higher the fecal cortisol levels, possibly due to cold stress (Takeshita et al. 2014). On balance, it can be concluded that Japanese macaques are well adapted to winter conditions, apart from those seasonal times when temperatures regularly drop below 0°C, during which time they experience a serious metabolic challenge from the cold (Thompson et al. 2017).

## 3 Nutritional Stress

Japanese macaques are fundamentally fruit and leaf eaters (Furuichi, Takasaki, and Sprague 1982). Their wide latitudinal and altitudinal distribution, as well as seasonal shortages of dietary items, result in episodic nutritional stress. Nutritional stress can take two, often synchronous, forms: undernutrition in the winter; and accompanying malnutrition through ingestion of secondary plant metabolites, particularly toxic compounds or anti‐feedants in fallback foods (Hanya et al. 2007; Hanya et al. 2006).

### 3.1 Undernutrition

Japanese macaques show dietary flexibility both latitudinally and seasonally. Those macaques which live in the more northerly cool temperate forest have larger home ranges, travel twice as far between food patches, have less than half the population density but twice the group size, spend much more time feeding, and eat much less fruit proportionally (Hanya 2010). Their favorite foods of high‐quality seeds and nuts are in short supply in the autumn when they must accumulate sufficient fat stores to survive the winter. Hamada and Yamamoto (2010) reported that commencing as young as 2 years, fat accumulation showed a striking circannual fluctuation, peaking in late autumn.

Japanese macaques at high altitude or latitude are judged to live in energy crisis during the winter because of poor food quality and deep snow (Nakayama, Matsuoka, and Watanuki 1999). On Kinkazan Island (38.3° N), daily energy and protein intakes in the winter were only a third of that in the summer, necessitating that they draw upon fat reserves (Nakagawa 1989). Energy intake of northern populations in the winter is only 60% of energy expenditure (Enari and Sakamaki‐Enari 2013). The time spent feeding in northern areas with deep snow, decreasing availability of food items and increasing energy costs (searching and thermoregulation), is 1.7 times that in the south (Agetsuma and Nakagawa 1998). Food scarcity on the Shimokita Peninsula is well documented (Hamada and Yamamoto 2010). Population density is second lowest of 11 populations throughout Japan (Hanya 2010). In their study of a Shimokita population (41.4° N, 140.9° E) who seasonally experienced “deep snow,” Nakayama, Matsuoka and Watanuki (1999) showed that juveniles fed mainly on dormant winter buds and tree bark. However, the main limiting factor on food intake was feeding rate (typically 2.5 h of their activity budget out of a 7‐h day) which yielded an energy intake 80%–88% of energy expenditure. They estimated that winter foraging conditions at Shimokita lasted about 100 days during which time individual juveniles drew upon fat reserves, nevertheless losing on average about 17% of annually maximum body mass. Another limiting factor for macaques is gut capacity. Due to reduced daylight hours in wintertime, macaques can only spend a maximum of 70% of their active time feeding; consequently, ingested amounts are often less than daily energy requirements, resulting in food deficiency (Hanya 2010). During the winter, the main foods such as mature leaves, dormant buds and bark are abundant, according to Hanya, not requiring them to roam far and thus conserving energy which they can put towards thermoregulation. So, while losing body mass in the wintertime, fat stores enable Japanese macaques to weather the season fairly well. Reasoning that dry fur with its entrapped air is a far better insulative layer than is fat (Grahn and Heller 2004), and that macaques can protect themselves from a degree of the cold through huddling (especially with their own young (Ueno and Nakamichi 2016) or sun‐bathing (Tsuji et al. 2008)), fat deposits are deemed to have more of a nutritional, rather than insulative, role (Hori et al. 1977).

### 3.2 Secondary Plant Compounds

Many plant species have evolved SPCs to discourage herbivory by insects and vertebrates (Agrawal and Weber 2015). They are also known as secondary metabolites and such chemical deterrents may number in the hundreds of thousands (Glander 1982; Kessler and Kalske 2018). SPCs, including flavonoids, alkaloids, terpenoids, and polyphenols (especially tannins) are ubiquitous in plants consumed by herbivores (Coley and Barone 1996), potentially affecting their physiology through toxicity or as anti‐feedants (Wrangham and Waterman 1983).

While we think that SPCs may be an important cause of enamel hypoplasia in nonhuman primates (Skinner et al. 2024), we emphasize that this has yet to be shown. Furthermore, whether exposure to SPCs, which may result in enamel hypoplasia, is deliberate (as in choosing to eat medicinally active plants) or adventitious (as in seasonal nutrient switching) has not been evaluated. Nevertheless, we draw attention to observations which suggest that the ingestion of SPCs should be considered as a potential cause of enamel hypoplasia in Japanese macaques. For example, vincristine, an alkaloid from the periwinkle plant introduced in vitro, produces enamel hypoplasia in hamsters and is predicted to do the same in human children (Lyaruu et al. 1995). Similarly, painkillers, derived synthetically from lignin in coal tar, taken during pregnancy produced enamel hypoplasia in the forming primary teeth of 38% of human infants (Miyamoto et al. 2023).

SPCs, particularly tannins, can reduce food intake in response to the unpalatability and astringency of tannin‐containing plants (Kumar and Singh 1984), or dietary tannins can reduce growth rates systemically through affecting post‐absorptive metabolism (Butler 1992). Alternatively, SPCs may be sought out by Japanese macaques for their antiparasitic or beneficial medicinal qualities (MacIntosh and Huffman 2010), only inadvertently affecting enamel formation.

“…hydrolysable tannin functions as a defensive chemical against folivorous animals, including both insects and mammals, whereas condensed tannin does so against microbes and fungi {}. In fact, leaf‐food selection by rhesus macaques was affected more strongly by hydrolysable tannins than by condensed tannins {}. Hydrolysable tannin content tends to show stronger seasonal fluctuations than condensed tannins, and the pattern varies with the types of molecules {}. Therefore, the presence of particular types of hydrolysable tannin, rather than the total amount, may affect leaf‐food selection in a particular season.

(Hanya et al. 2007)

Yakushima Island, in the more temperate far south of Japan, is home to coastal and higher inland populations of monkeys. At Yakushima, coniferous leaves contain large amounts of condensed tannin and Japanese macaques have not been observed to eat these leaves (Hanya et al. 2011). Feeding times on mature broad‐leaved deciduous foliage is only 5% for the coastal versus 38% for the inland group who are, consequently, at a much higher risk of consuming large amounts of condensed tannins (Hanya et al. 2007).

Japanese macaques from further north are said to select mulberry winter‐dormant buds as a fallback food since they are comparatively low in tannins (Enari 2014; Enari and Sakamaki 2010; Enari and Sakamaki‐Enari 2013). Later in the winter season, they will turn to eating the outer bark (Watanuki et al. 1994), which is much higher in condensed tannins (1%–3.5%) (Ishii and Ohara 2004). While mulberry is the preferred winter diet of Shimokita Peninsula macaques (being foraged twice as frequently as any other tree species), the buds and bark which they consume heavily for about 8 weeks, do not always qualify as an adequate diet in terms of energy intake versus foraging efficiency (Watanuki et al. 1994). Interestingly, for unknown reasons, the macaques feed more often on shade‐intolerant plants, such as mulberry (Enari 2014; Sakamaki et al. 2011). It may be that feeding in the sunlight to keep warm is preferable; however, it is well known that light intensity typically increases plant secondary metabolites (Mole, Ross, and Waterman 1988). Uniquely, Kinkazan Island macaques show a strong dependence in winter on the bark of *Zanthoxylum piperitum* (Japanese pepper) which is thorny, smelly and unattractive to deer (Tsuji and Takatsuki 2004) and inhibits food intake in rats (Epple et al. 2004). Exposure to higher levels of SPCs need not occur just in the winter since spring leaves are high in phenolic content; for example, peaking in May in *Quercus acutissima* (sawtooth oak) (Ito and Hayashi 2020) made tolerable, the authors suggest, by high sugar content. In Yakushima, the animals often fed on immature, unripe fruit (Maruhashi 1980), which is often higher in tannins (Wrangham and Waterman 1983). Japanese chestnut, consumed by these monkeys, contains abundant phenolic compounds (Tuyen et al. 2017). In short, Japanese macaques respond to seasonal variation in exposure to tannin concentration, lasting many weeks, through selective feeding and diet switching.

We have reviewed thermo‐regulatory and two forms of nutritional stress in Japanese macaques which they could experience together or separately. Importantly, each is seasonal in terms of timing and duration and thus could be linked to the repetitive nature of LEH, which is the focus of our study. It is worth noting that the immature Japanese macaque would have to be experiencing either sustained near 0° C temperatures and/or serious fat depletion or, alternatively, experiencing sustained negative exposure to plant metabolites, in order for enamel hypoplasia of observable duration to be created. Additionally, current perspectives on developmental stress among Japanese macaques are based on observations of mostly adults, not infants and juveniles who are still growing their bodies and teeth and who cannot rely on being fed by their mother in the food‐scarce season and among whom mortality is high (Hamada and Yamamoto 2010). Weaning occurs relatively early in this primate, usually within the first year of life commencing about age 6 months. They show rapid development of independent feeding (Hamada and Yamamoto 2010), having been observed to lick or chew solid objects such as twigs as early as 2 weeks and eating some twigs by 5 weeks. Thus, it is expected that LEH events would be present in teeth that form during this life history stage. As the large canine crown of male Japanese macaques begins forming during early infancy and crown formation lasts approximately four or 5 years, this tooth is an ideal tooth to investigate the potential causes of rLEH in this species.

## 4 Methods

### 4.1 Ethical Note

Japanese macaques are numerous on the islands of Japan, numbering in excess of 110,000 individual animals that occupy a range of habitats (Fooden and Aimi 2005). They are judged to be a species of “least concern” by the International Union for Conservation of Nature, although there are locally threatened and extirpated populations. Specimens examined in this study consist only of skeletal remains from the Center for the Evolutionary Origins of Human Behavior and access was granted through EHUB application processes. Mao Asami created high resolution dental molds at the Institute. Dental thin sections were made by Akiko Kato; no hard tissues were transported internationally. This research was approved by the Center for the Evolutionary Origins of Human Behavior (EHUB) and conforms to the American Society of Primatologists Principles for the Ethical Treatment of Nonhuman Primates.

### 4.2 Study Population

**FIGURE 1:**
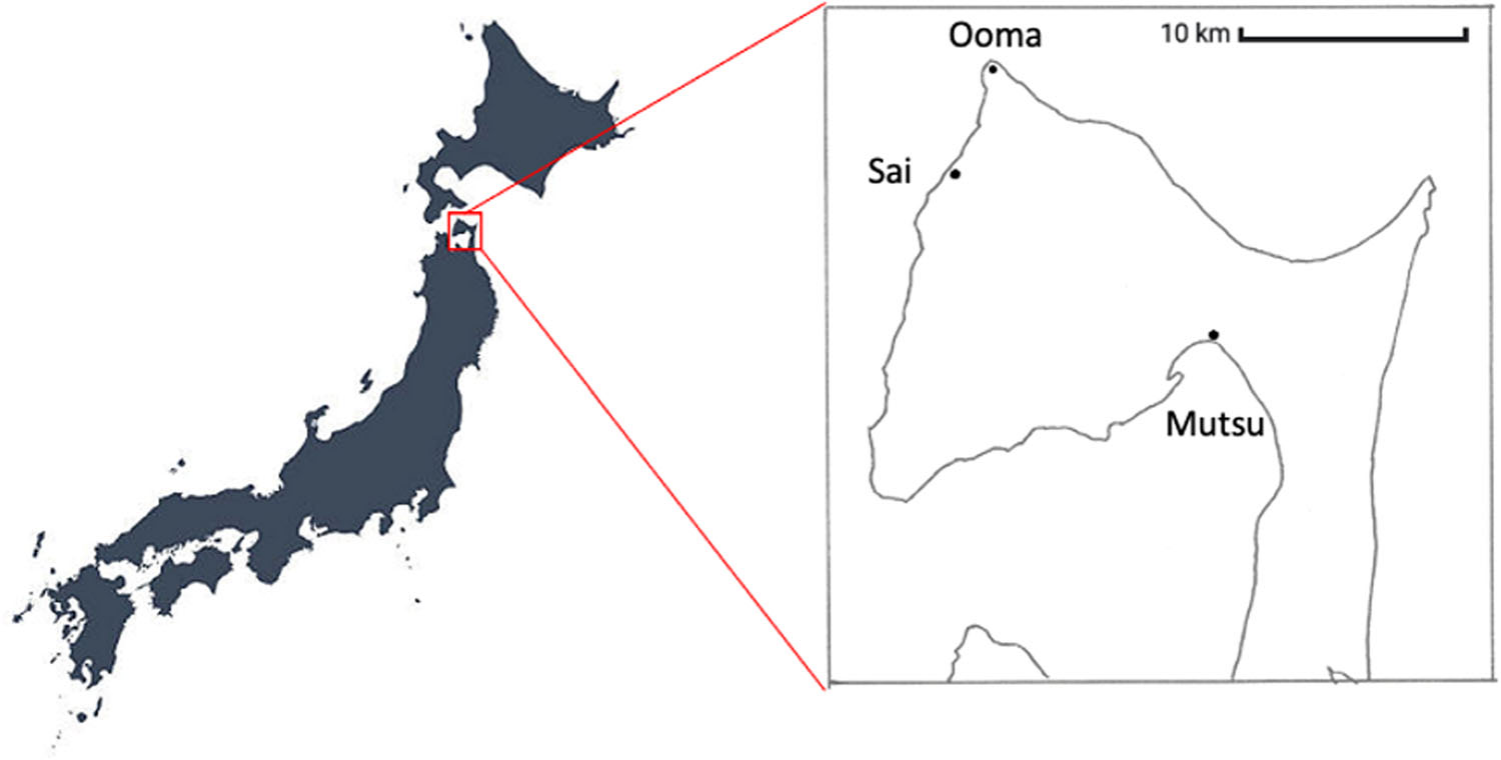
Shimokita Peninsula, Honshu Island, Japan. Mutsu is the location for monkeys as well as weather data.

In that our earlier studies of prevalence of LEH in nonhuman primates (e.g., (Guatelli‐Steinberg and Skinner 2000; Hannibal and Guatelli‐Steinberg 2005; Skinner 1986)) have not elucidated etiology, we felt that documentation of incidence (i.e., temporal occurrence) of LEH among Japanese macaques, known for marked seasonal variation in food and nutrition, might help to understand causation. Because of the heightened seasonality and deep winters that prevail in northern Japan, we chose to initiate a pilot study of the potential presence, timing and severity of LEH among recent wild monkeys (*Macaca fuscata fuscata*) from the Shimokita Peninsula, Honshu Island (latitude 41.4° N) Japan (Figure 1). In a wide‐ranging study of geographic variation in Japanese macaques, the most different population was that of Shimokita (Kawamoto et al. 2008), where they form a geographic and genetic isolate (Kawamoto 2010). As such, we acknowledge that our sample may not be representative of the physiological experiences of monkeys living in more temperate latitudes.

### 4.3 Data Collection

rLEH is a remarkably common developmental defect among New and Old World monkeys (Chollet and Teaford 2010; Guatelli‐Steinberg and Benderlioglu 2006; Guatelli‐Steinberg and Skinner 2000; O'Hara and Guatelli‐Steinberg 2020) and, especially, apes (Guatelli‐Steinberg 2000; Guatelli‐Steinberg, Ferrell, and Spence 2012; Skinner 1986, 2021; Skinner, Dupras, and Moya‐Sola 1995; Skinner and Hopwood 2004; Skinner and Pruetz 2012; Skinner and Skinner 2017). Our focus in this study is on whether regular recurrence prevails in the individual, and on its timing, not on how common the pattern may be in a population. Whether prevalence is 15%, 50% or 85% in a primate sample, for example, will not enable the investigator to identify the nature of an underlying cause. High prevalence of rLEH within taxa, and wide‐spread occurrence taxonomically, may reflect that EH is a generalized response to a host of different, largely independent stressors that impinge on a juvenile primates' tissues as they mature; or there is an underlying cause common to most occurrences that is experienced by a wide variety of nonhuman primate populations and taxa. As Japanese macaque populations are distributed over a large eco‐geographical range, are already are so well‐studied, for example (Fooden and Aimi 2005; Hamada et al. 1999; Maruhashi 1980; Nakagawa 1989; Tsuji et al. 2015) and that their developmental environments can be manipulated under carefully provisioned conditions (Aoki et al. 2015; Landi et al. 2021), they afford an ideal primate sample to test these alternatives. However, before one embarks on so ambitious a series of projects, it must first be demonstrated that a pattern of regularly recurrent episodes of LEH occurs in Japanese macaques at all. If this can be established in the present study, we can then move on, in the future, to examine the effects of socio‐sexual and ecological environments among a variety of Japanese macaque populations.

**FIGURE 2:**
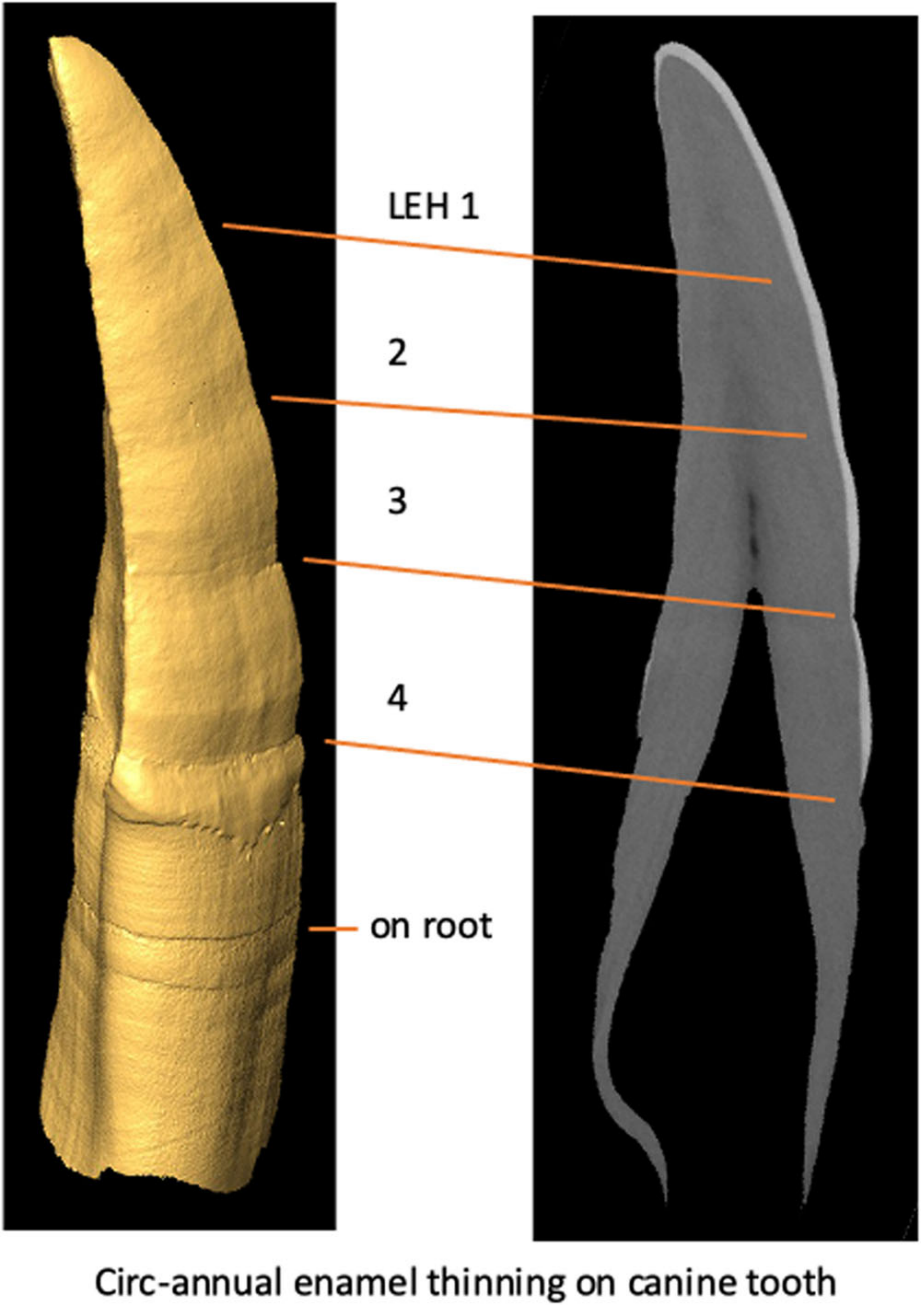
Linear enamel hypoplasia (LEH) in a male Japanese macaque upper canine tooth (EHUB 10077 CT images from MMS). Note how little the dentin is affected compared to the enamel (right panel), although it is clear that there is continued repetition of distress expressed on the root dentin (left panel).

It was clear upon preliminary examination of *M. fuscata fuscata* specimens in the curated collections at EHUB that LEH was present (Figure 2) among individuals of either sex. As the focus of our study is on the number and timing of LEH within individuals (and not of prevalence in the species as a whole), upper canines from 15 male individuals (Table 1) were preferentially chosen because of their large crown size (and presumably longer formation times (Schwartz, Reid, and Dean 2001)) and hence greater potential for recording stressful events within the time that individual crowns take to form.

**TABLE 1:** The sample of male maxillary canine tooth casts from Shimokita, Aomori Prefecture, of wild Japanese macaques (EHUB).

EHUB ID	Source	Date of death	Maturity	Body weight (kg)	Crown height (mm)	Side	LEH per crown	Thin‐sectioned
9822	Sai	2012‐08‐23	“Child”	9.09	21.9	Right	4	Yes
9823	Sawamutsu	2012‐11‐28	Sub‐adult	10.56	19.8	Left	5	
9828	Mutsu	2012‐08‐11	adult	13.04	20.3	Right	4	Yes (RP)^a^
9858	Mutsu	2012‐09‐04	Sub‐adult	8.13	22.1	Right	4	Yes
9861	Sai	2012‐08‐03	Sub‐adult	11.34	20.2	Right	4	Yes
10074	Mutsu	2013‐02‐23		9.98	20.5	Right	5	Yes
10077	Mutsu	2012‐11‐07	Sub‐adult	11.59	21.4	Right	4	
10081	Ooma town	2012‐02‐27	Sub‐adult	7.75	18.2	Left	5	
10088	Mutsu‐chi	2013‐02‐22		9.67	20.2	Right	5	Yes
10092	Ooma‐town	2012‐11‐13	Sub‐adult	13.47	18.9	Right	5	
10097	Mutsu	2012‐12‐07		12.18	21.2	Right	5	Yes (RP)
10654	Mutsu	2013‐12‐22		10.48	19.2	Left	4	Yes (RP)
10655	Mutsu	2014‐01‐21		9.04	20.1	Left	4	Yes
10681	Ooma‐town	2014‐11‐16		11.84	22.6	Right	4	Yes (RP)
10683	Ooma‐town	unknown		12.03	18.6	Right	3	Yes (RP)

^a^ Retzius periodicity

As the animals came from the wild between 2012 and 2014, with already erupted canine teeth that take several years to form, they probably commenced canine crown formation (and LEH) at an indeterminate date around 2005 and later. The study sample originates in the Aomori Prefecture, Japan. We obtained modeled meteorological records (www.meteoblue.com) for Mutsu City, Aomori Prefecture, at latitude 41.3°C, on the Shimokita Peninsula. Meteoblue's earliest weather data for the specified location commences in 2015, while our animals died a few years earlier.

## 5 Retzius Periodicity (RP)

### 5.1 Recording the Outer Enamel Surface

Dental crowns grow from the tooth tip in a cervical direction. Enamel is secreted by ameloblasts along an enamel prism with a circadian rhythm accentuated by an episodically slower rate of enamel formation recurring every few days or so (optically visible in thin section). These long‐period Retzius lines represent the forming enamel surface at different stages of enamel thickening and crown formation (Dean and Scandrett 1996). Where Retzius lines meet the outer enamel surface, they outcrop as tiny furrows in the imbricational enamel creating, between successive lines, tiny ridges termed perikymata. The number of cross striations between successive Retzius lines, visible in a microscopic thin section of the enamel is referred to as their periodicity (Reid and Ferrell 2006). RP is thought to be invariant within the permanent teeth of an individual, but see (Mahoney et al. 2016; McFarlane et al. 2021), although can vary within and between taxa (FitzGerald 1998). Late secretory ameloblasts are particularly sensitive to developmental stress (Kierdorf et al. 2021) resulting in horizontal furrows in the outer enamel surface that may be easily seen with the naked eye or at low magnification (Boyde 1970). Perikymata within and between hypoplastic furrows of thinned enamel can be counted and, knowing RP, converted to days to estimate the timing and duration of episodes of distress in the young primate (Goodman and Rose 1990; Hillson 2014).

High‐resolution molds were taken of the labial surfaces of little worn upper canine teeth, selected for visibility of LEH, spanning from the occlusal tip to the cervical margin, including a small portion of root. Casts were made with low‐viscosity epoxy casting resin. To observe perikymata, casts were imaged with a Hitachi S‐2600N scanning electron microscope (SEM). Sputter‐coated casts were tilted at 30°, with the cusp tip down relative to the detector, to enhance visibility of perikymata, and photographed at 30× magnification. Anywhere from 10 to 25 SEM photographs per crown were combined in a high‐resolution photomontage created with Adobe Photoshop Elements 19 for counting of perikymata within and between LEH furrows (where possible) (Supporting Information S1: Images file).

### 5.2 Thin Sectioning Protocol to Derive Retzius Periodicities

RP is the key to deciphering the timing of enamel defects. Hitherto, there has been only one study of RP in Japanese macaques (7 days) and no indication of the number of animals studied (Fukuhara 1959). Confusingly, Fukuhara's Fig. 12 (thin section of a tooth) states (in translation): “Taiwan Monkey, Macacus fuscatus, upper jaw, the first premolar, the angle between striae of Retzius and dentino‐enamel junction is 35°, 60 times (magnification)”. Conceivably, Fukuhara's RP of 7 days is based on a hybrid of an introduced Formosan rock macaque and a Japanese macaque. Notably, Fukuhara's Retzius periodicities (7–8 days) for macaques *(M. rhesus (mulatta), M. fuscatus (fuscata), M. cyclopis, M. cynomolgus (fascicularis))* are higher than those reported by anyone else including *M. nemestrina*: 4–6 days (Bowman 1991; Guatelli‐Steinberg et al. 2009; Kufeldt and Wood 2022; Okada 1943). Consequently, we created new thin sections from select teeth in our study affected by LEH (Table 1).

Before histological sectioning, the entire tooth was CT‐scanned using microtomography (Rigaku CosmoScan GX). The resulting 3D model was used to orient the tooth before selecting the sectioning axis. A line perpendicular to the perikymata passing through the apex and the maximum labial cervical enamel extension was used as the cutting line. Once the cutting line in the labio‐lingual direction was determined, a grinding surface, 1.5 mm distal to the cutting line, was selected. The tooth embedded in methyl methacrylate block was then sectioned labio‐lingually using an IsoMet Low Speed Saw (Buehler). The section was lapped on a grinding machine using 45, 30, 15 and 6 μm diamond disks, ultrasonicated, and finished using a 1 μm alumina suspension. This face of the section was then fixed to a microscope slide under pressure using UV Resin (Logitech Ltd.). After curing, the section was lapped to a thickness of < 100μm, ultrasonicated, and again finished using a 1 μm polishing suspension. The section was once more ultrasonicated and then cleaned with alcohol. Each section was observed with a phase contrast microscope (Keyence BZ‐X710) and polarized light microscope (Eclipse E200, Nikon Solutions Co. Ltd.). Five sections yielded results. Daily enamel increments between striae of Retzius are indicated in our resulting figures with contrastingly colored arrows. Cross‐striations counts were made independently by AK and MFS.

### 5.3 Data Analysis: Counting and Measuring

Data were analyzed with IBM SPSS29. Raw data are supplied in Supporting Information S2: Table 1. Tests of differences in non‐normally distributed dimensions between ordinally scored LEH salience and among numbered LEH (e.g., LEH 1 vs. 2 vs. 3) were made with non‐parametric Kruskal–Wallis test. In all tests, precise probability levels are provided.

To ensure consistency of homologizing defects among image types (naked eye, macrophotographs, digital microscopy and scanning electron microscope (SEM)), defects on sputter‐coated casts were visualized initially with a hand lens at 10× under oblique lighting and then recorded with a Canon EOS M50 macrolens. Perikymata counts are essential for our study as we use them to (a) directly predict duration and interval between enamel furrows, and (b) calculate average perikyma width to predict likely perikymata counts in other parts of dental crowns where perikymata are not sufficiently visible. In such instances, where perikymata were too worn to count, it was necessary, to determine the “interval” between LEH as well as “duration” in time of discrete LEH, to use an estimate of the number of perikymata expected over a given length of the upper canine crown. We used two ways to derive perikymata counts in the occlusal wall (i.e., duration): firstly, on the SEM images, looking perpendicularly at the surface and counting them directly in what is, in effect, a flattened image of the LEH with uncertain boundaries; and, secondly, using an Olympus confocal LEXT OL3100 microscope, looking at a profile of the enamel furrow from the side, where the perikymata are not easily seen, but where the onset width and deepest point are easily measured, enabling estimation of perikymata count in the occlusal wall from average perikyma width in a particular region. Onset refers to the enamel measure during the first phase of experienced stress while the whole width and total area of missing enamel include the recovery phase, inferably after the episode of stress has ceased.

Use of this instrument and replicability of measures have been evaluated for Japanese macaques (Skinner 2023).

In our study, the distance between perikymata (hereafter termed perikyma width) changes during tooth development and typically increases from the tip of the canine to the cervix in this species. We first divided each canine crown into quarters by dividing the total crown height by four and naming these occlusal, second, third and cervical quarters, respectively. We then measured the overall width of the included perikymata in each region of the crown in each individual tooth, where visible. These widths were used to calculate the median and mean width of perikyma for each region, and medians were used in the calculation of the estimated time to create an LEH. To capture the interval between LEH defects, scaled macrophotos of successive LEH intervals (typically deepest point to deepest point, visually, of adjacent furrows) were taken with a Keyence digital microscope. The calculated median perikyma width for the quarter of canine crown height containing most of an inter‐LEH interval was used to calculate the number of perikymata between onsets of successive LEH.

We compare the observed and predicted number of perikymata between and within LEH defects in Table 2. Among a total of 68 recorded LEH, there are only 4 intervals and 14 durations for which perikymata could be observed directly. For both, on average, there is less than one perikyma difference between observed and predicted values; nevertheless, for discrete LEH the differences can be substantial as may be seen in Table 2.

**TABLE 2:** Comparison between “observed” and “predicted” number of perikymata between and within^a^ episodes of enamel hypoplasia.

		Interval		Duration	
Individual	LEH number (or interval)	Observed	Predicted	Observed	Predicted
9822	1 (to2)	79	63	7	9.4
9828	1			4	3.7
	2			11	8.6
9858	1			6	7.4
	2			7	15.4
9861	1			10	8.9
	3			11	10.8
10077	4			16	7.7
10654	1			5	3.2
10655	1	43	54	5	3.3
	2	55	64	9	8.3
	3	33	29	10	10.5
	4			8	7.7
10681	4			15	8.3
Mean		52.5	52.5	8.9	8.1

^a^ Occlusal wall only.

### 5.4 Determining Cyclicity Through Ratio Analysis

In this approach, we compare the ratio of perikymata count for a particular interval divided by the perikymata count in the preceding interval on the same tooth. The advantage of this technique, which relies on the reasonable assumption that RP remains constant within a tooth (Dean 1987; FitzGerald 1998), is that the potential for different Retzius periodicities, among aggregated teeth, is obviated (Skinner et al. 2024). This method can show unequivocally the regularly recurrent nature of stressful events, if most ratios center on 1.0 (0.0 when expressed as natural logs to normalize the distribution [Skinner and Ji 2024]).

### 5.5 Measuring Salience of LEH

Enamel furrows are not considered a direct measure of “stress” (Hillson 2017). Rather, enamel depressions are a proxy for stress as mediated not only by enamel formation geometry (which can vary among teeth, sexes and taxa) (McGrath et al. 2019) but, also, by how an individual animal's physiology (e.g., frailty) and behavior (e.g., foraging efficiency) affect enamel matrix secretion. Our first approach to measure LEH was to score the visibility of a defect on an ordinal scale from “none” through “faint or mild” to “marked”; a procedure having the advantage of evaluating the entire LEH mesio‐distally around the crown's labial surface. One defect was very broad without clear boundaries which was designated a “swale.” Visibility of an LEH is a function of both width and depth, since it is known that sustained distress produces deeper defects, because of the geometry with which enamel is deposited (Guatelli‐Steinberg 2003). Thus, all else being equal, more *severe* distress produces deeper defects with a steeper angle of descent (Skinner and Skinner 2017), while *sustained* distress produces wider (and deeper) defects. The metrical assessment of enamel furrows is relatively new (McGrath et al. 2018; McGrath et al. 2021; Skinner et al. 2024; Skinner and Skinner 2017) and needs many more applications of the instruments. The relationship between defect depth and duration necessitates a means of controlling for time, in both depth and width, so as to evaluate the degree of enamel thinning. Thus, a second, more objective approach using confocal microscopy, is to measure the amount of missing enamel scaled by daily secretion rate. This measure of relative distress can be applied across analytical categories (e.g., teeth, sexes, populations, taxa). Termed “enamel deficit ratio,” the index of enamel thinning is the ratio of “daily deficit in depth” to “daily secretion rate” (Skinner 2023). Daily secretion rate was measured along enamel prisms between striae of Retzius visible in thin sections.

### 5.6 Estimating the Chronological Age of an Individual When Stress Events Occur

The final aspect of dental formation that is essential for understanding the timing of LEH defects is the onset and completion of the sexually dimorphic macaque canine crown. The literature is almost silent on this phenomenon. A related taxon, *Papio cynocephalus* showed canine crown initiation at 0.9 years in both sexes but crown completion at 2.0 years in females compared to 3.1 years in males (Swindler and Meekins 1991). In a small sample of *M. irus* upper and lower canines, an average eruption occurred by 3.5–4.0 years. Based on the relationship between crown completion and eruption in their incisors, canines in this species likely completed their crowns approximately 1 year earlier than their eruption, that is, 2.5–3.0 years (Bowen and Koch 1970). Another study suggested that, at the very outside, crown completion among three species of macaque was accomplished in 3.8 years (Guatelli‐Steinberg et al. 2009). However, in a more detailed study of variation in canine mineralization based on a compilation of laboratory collections of rhesus monkeys (*n* = 13 to 33 animals, sex unspecified), Trotter, Hixon and MacDonald (1977) report the following:

**Table:** 

	Onset (years)	Completion (years)
Upper canine	Median 1.02 (0.48–2.73)	1.94 (0.34–3.96)
Lower canine	Median 1.02 (0.75–3.72)	1.85 (0.34–3.42)

**FIGURE 3:**
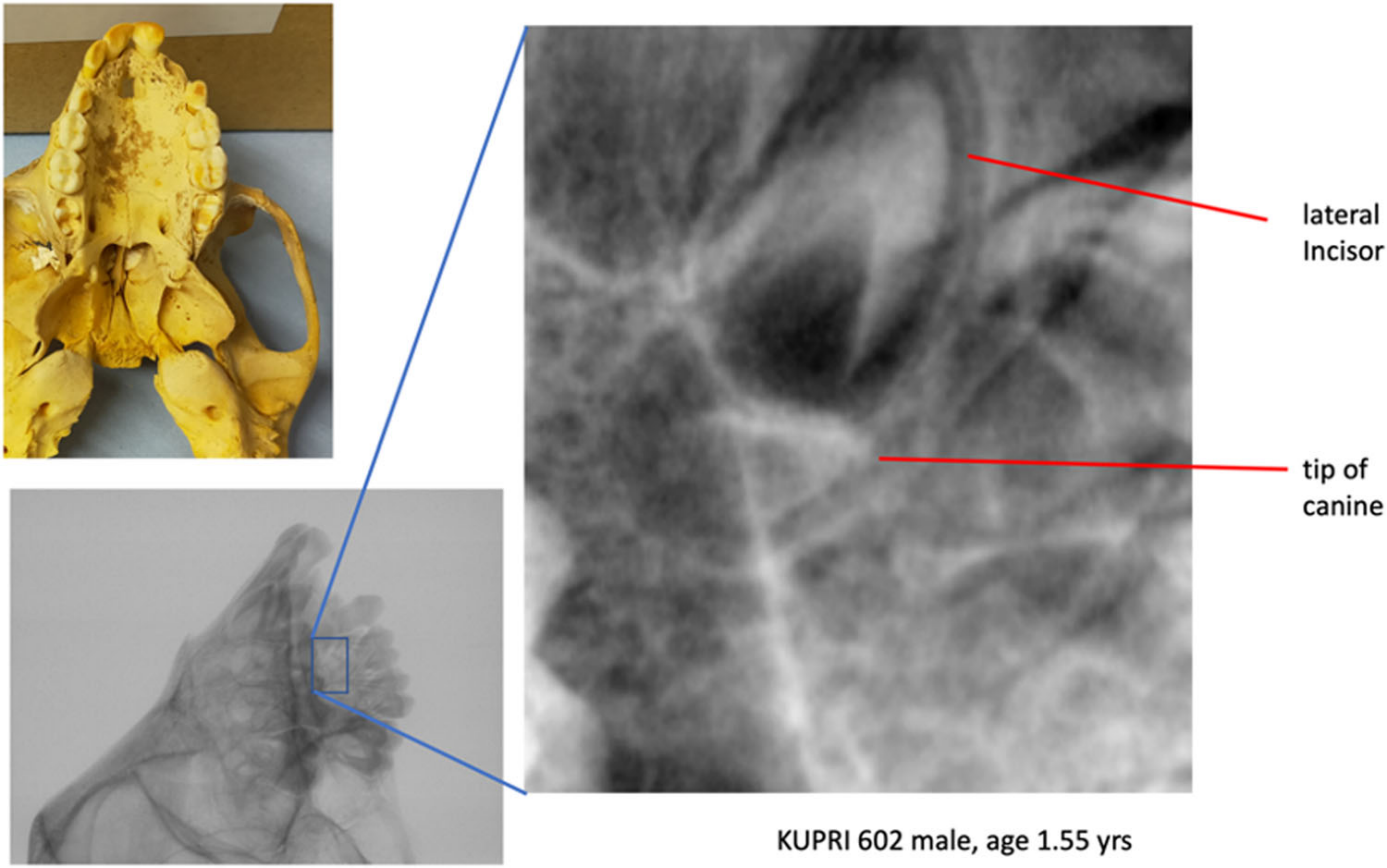
Onset of maxillary canine crown formation.

There are no comparable data for Japanese macaques, to our knowledge. Consequently, we include here a radiograph of an infant male (EHUB 602) aged 1.55 years who shows just the beginning of mineralization of the canine tip (Figure 3), consistent with the rhesus data (Trotter, Hixon, and MacDonald 1977). Thus, if typical, any stressful events occurring younger than about a year and a half old will not be recorded in the canine tooth. Since we observe four or five of what we construe to be circ‐annual LEH on the male canine crowns in this study, crown completion extends to much older ages than those provided by Trotter for this stage, but somewhat more compatible with gingival eruption ages for Japanese macaque upper canines from Ariyashima West colony: “not before age 5 years in males, a full year later than females” (Nass 1977).

### 5.7 Predictions

In that the only prior study of enamel hypoplasias in Japanese macaques that addressed etiology, attributed specifically “large plane form defects” to anthropogenic forces (Towle et al. 2024), our null hypothesis is that LEH observed in this study will be aperiodic. If numerous LEH are observed, we predict that the inter‐LEH interval will recur seasonally, and the duration of repeated LEH will be consistent with the duration of winter conditions and/or exposure to SPCs.

## 6 Results

### 6.1 Finding Retzius Periodicity

**FIGURE 4:**
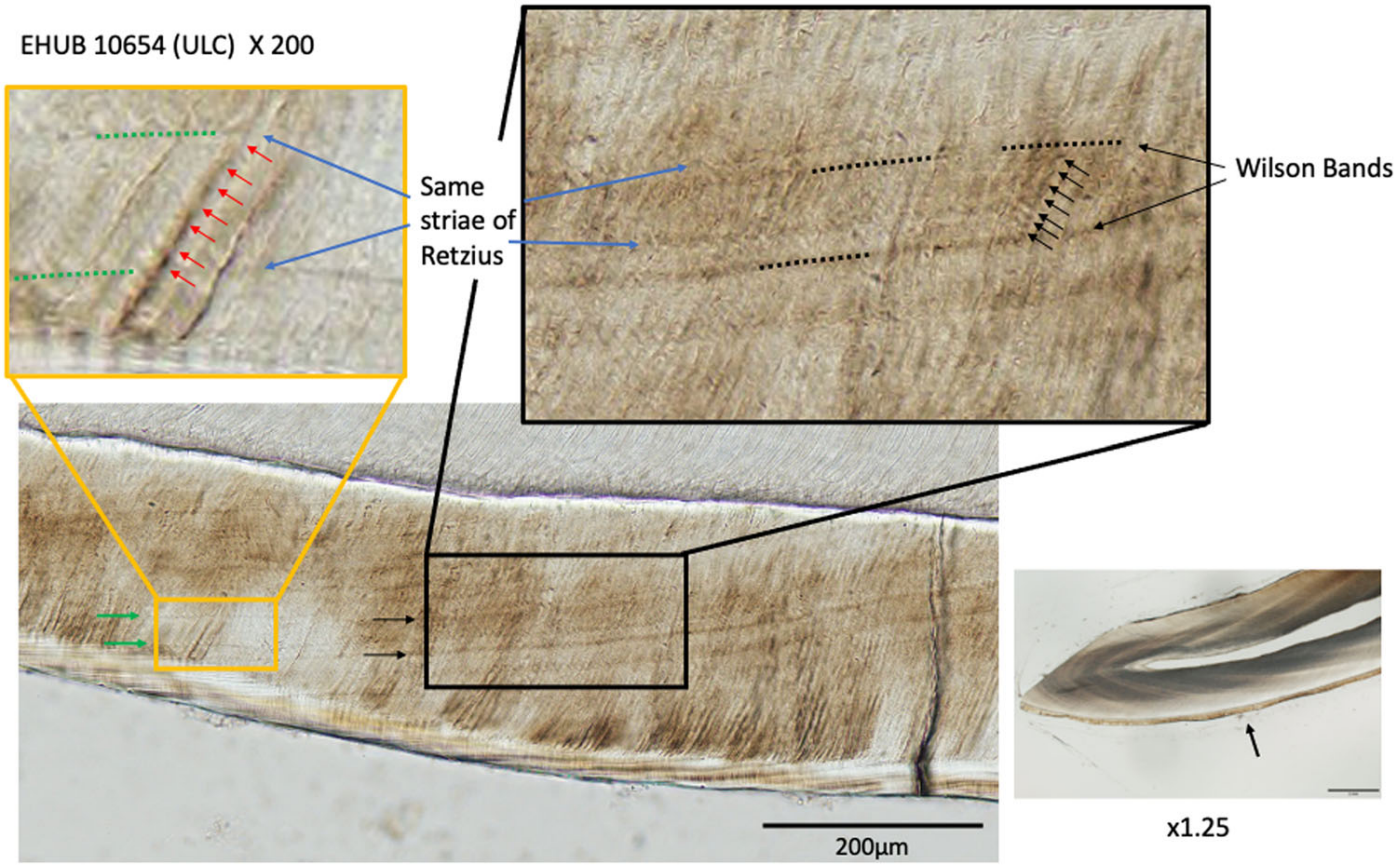
EHUB 10654 (ULC). Retzius periodicity = 7. This instructive example shows the contrast between striae of Retzius (faint and tending to be visible near the outer enamel surface‐yellow box) versus Wilson bands (darker and tending to course from the EDJ to the OES) which here are about seven days apart. Daily increments of enamel are shown by red arrows or, in the box on the right, in black.

We provide estimates of RP from thin sections. One of us (AK) worked with original slides while another (MFS) used digitized images made from the originals. We performed our counts independently. The result for one tooth is shown in Figure 4. The RP is 7 days for all five specimens (with the possible exception of EHUB 10097 for whom RP may be 8).

### 6.2 Interval Between Recurrent LEH: Combined Teeth

**FIGURE 5:**
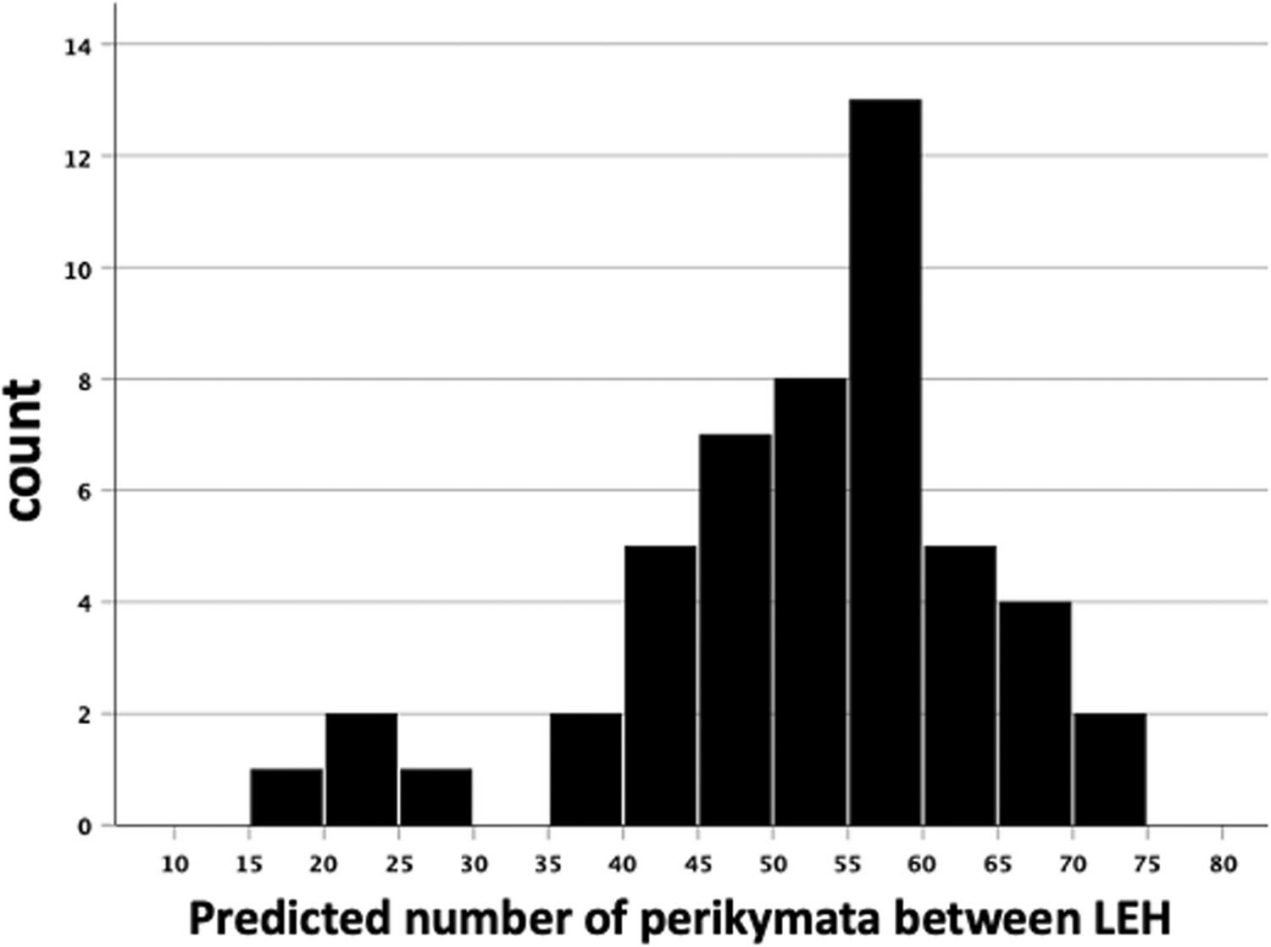
Frequency distribution of predicted number of perikymata within measured space between adjacent LEH (see text for further details). LEH, linear enamel hypoplasia.

An estimate of LEH furrow recurrence can be found by counting the number of perikymata between successive LEH and multiplying by a RP of 7 days. Unfortunately, due to labial wear of the outer enamel surface, this was only possible for four pairs of adjacent LEH from two individuals (9822:79PK; 10655: 43, 55, 33PK). There is an average of 52.5 ± 19.8 perikymata for these directly observed four LEH intervals, which translates into a mean of 1.01 ± 0.38 years between each pair from these four events. Alternatively, we can calculate the likely number of perikymata between stressful events based on the distance separating them, allowing for variation along the crown in perikymata spacing (Figure 5).

Most intervals cluster around 50+ perikymata with an additional small group of narrowly‐spaced intervals. Table 3 presents the average time in years for aggregated teeth between stressful events in the occlusal, second, third and cervical quarters of the crown. The location of the LEH interval is allocated to that quarter in which the majority of the interval was situated. The predicted number of perikymata in successive quarters of the crown is very similar, but lower in the cervical quarter. The overall mean predicted number of perikymata between LEH is 52.5 ± 12.2, which translates to a recurrence interval of 1.0 ± 0.23 years.

**TABLE 3:** Predicted mean number of perikymata between adjacent LEH, based on median perikyma width in each quarter of crown height. Also shown is the estimated recurrence in years.

	Number of intervals	Predicted number of perikymata	Recurrence in years
LEH location		Mean (SD)	
Occlusal quarter	8	57.1 (10.3)	1.10
Second quarter	16	58.9 (7.7)	1.13
Third quarter	16	53.3 (7.9)	1.02
Cervical quarter	10	37.1(13.0)	0.71
Total/mean	50	52.5 (12.2)	1.00 ± 0.23

Abbreviation: LEH, linear enamel hypoplasia.

### 6.3 Cyclicity of LEH Intervals From Ratio Analysis: Single Teeth

**FIGURE 6:**
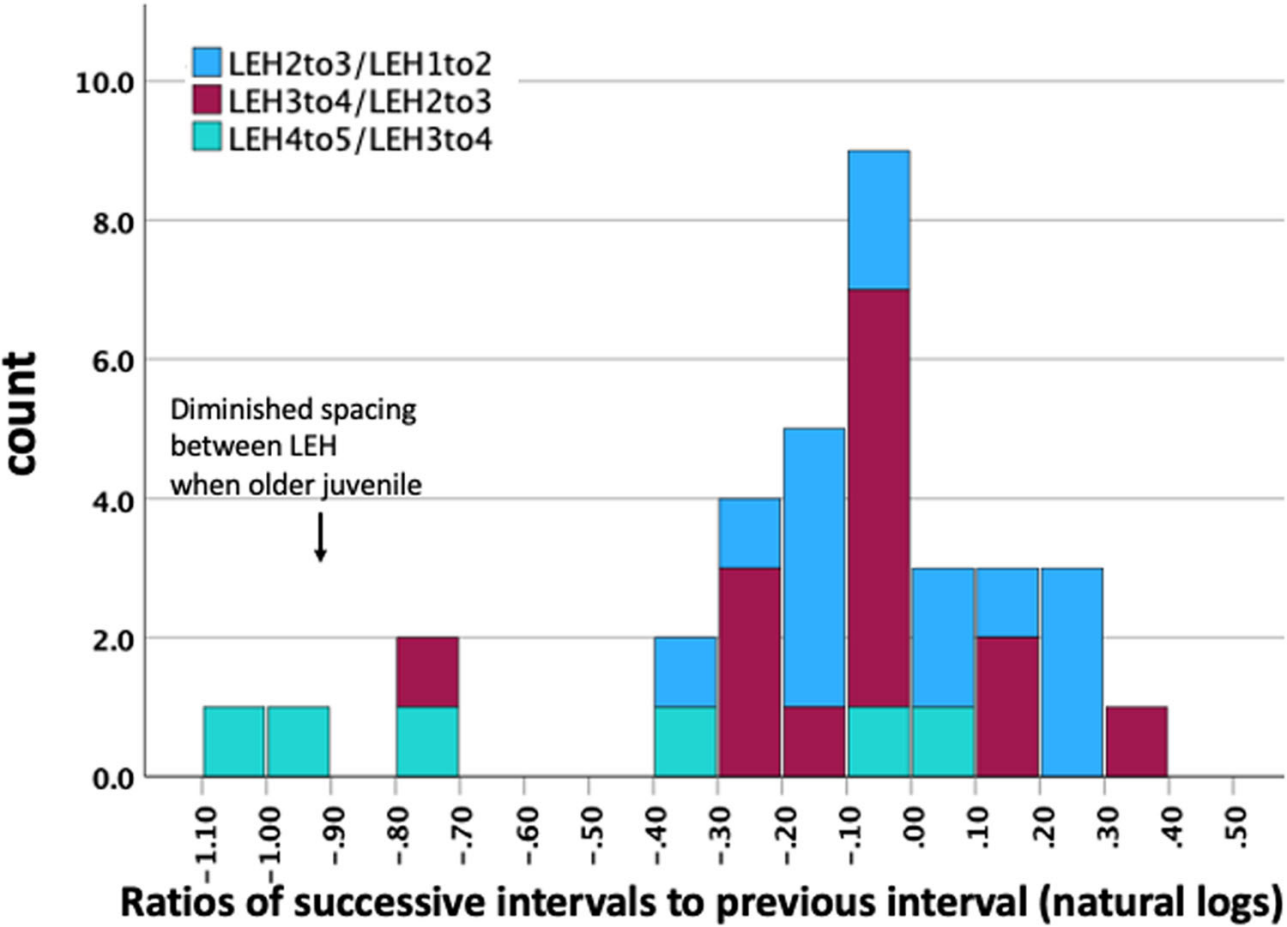
Frequency distribution of the ratio of perikymata count for an LEH interval to that of the preceding interval. Two groups are evident. LEH, linear enamel hypoplasia.

The utility of ratio analysis for detecting regular recurrence from inter‐LEH perikymata counts is that the effect of potential differences in RP between individuals is obviated. If the perikymata count for each succeeding interval is much the same as that for the preceding interval, then one could infer that a ratio of the two, would be close to 1.0. Figure 6 shows the results of this approach where ratios are expressed as natural logs to visually normalize the distribution (Skinner and Ji 2024). It can be seen that while this scenario is supported, there is a small group of intervals around a natural log ratio of −0.9. These four intervals occur close to crown completion in the last quarter.

As an alternative approach to the analysis of LEH recurrence, Table 4 reports the deviation of each successive interval's perikymata count from the mean LEH interval for that individual. Examination of the table reveals a number of quite low intervals (individuals 9823, 10088, 10092, 10655), which affect mean deviations for the individual and overall. The effect of these anomalous values is evident in Figure 6; they are evaluated in the Discussion.

**TABLE 4:** Deviation of perikymata (PK) counts of inter‐LEH intervals within each tooth from the mean inter‐LEH interval measured in perikyma for that tooth.

	Calculated inter‐LEH interval					Deviation from tooth mean				Mean absolute deviation (PK)
Ind	1–2	2–3	3–4	4–5	Mean	1–2	2–3	3–4	4–5	
9822	63	53	40		52.0	−11.00	−1.00	12.00		8.00
9823	57	70	55	21	50.8	−6.20	−19.20	−4.20	29.80	14.85
9828	47	40	46		44.3	−2.70	4.30	−1.70		2.90
9858	60	60	55		58.3	−1.70	−1.70	3.30		2.23
9861	56	51	47		51.3	−4.70	.30	4.30		3.10
10074	74	73	58	57	65.5	−8.50	−7.50	7.50	8.50	8.00
10077	58	47	43		49.3	−8.70	2.30	6.30		5.77
10081	42	54	45	47	47.0	5.00	−7.00	2.00	.00	3.50
10088	61	55	51	18	46.3	−14.70	−8.70	−4.70	28.30	14.10
10092	58	50	56	25	47.3	−10.70	−2.70	−8.70	22.30	11.10
10097	44	56	54	39	48.3	4.30	−7.70	−5.70	9.30	6.75
10654	59	61	58		59.3	.30	−1.70	1.30		1.10
10655	54	64	29		49.0	−5.00	−15.00	20.00		13.33
10681	70	49	68		62.3	−7.70	13.30	−5.70		8.90
10,683	68	60			64.0	−4.00	4.00			4.00
Mean	58.1	56.2	50.4	34.5	53.0 ± 7.0					7.2

Abbreviation: LEH, linear enamel hypoplasia.

### 6.4 Duration and Salience of Discrete LEH

In Table 5, we evaluate five LEH median measures (onset angle, depth, onset width, whole width and total area of missing enamel) taken with confocal microscopy.

**TABLE 5:** Measures (median) of LEH salience compared to assigned scores.

		Measurement				
Salience	N	Onset angle (°)	Depth (µm)	Onset width (µm)	Whole width (µm)	Total area missing enamel (mm^2^)
Faint	10	2.67	37.8	530.8	1035.0	0.0176
Mild	40	3.15	39.5	686.2	1453.8	0.0273
Marked	17	5.32	62.6	547.3	1555.0	0.0538
Kruskal–Wallis *H* *P*	67^a^	6.494	8.833	0.906	1.794	5.911
		0.039	0.012	0.636	0.408	0.052

Abbreviation: LEH, linear enamel hypoplasia.

^a^ There are 65 LEH plus two additional “defects within defects” included for measurement.

It can be seen in Table 5 that LEH, subjectively scored as increasingly salient (from “faint” to “marked”), are significantly deeper with a steeper onset angle and more missing enamel but do not differ in onset width.

A comparison of successive LEH along the developing crown (Table 6) found only one barely significant difference: LEH 3 (mid‐crown approximately) is wider on average than the rest.

**TABLE 6:** Measures (median) of LEH salience compared among LEH from near cusp tip to cervix.

					Area of missing enamel (mm^2^)	Predicted perikymata count in occlusal wall of defect	
LEH	Onset angle (°)	Depth (µm)	Onset width (µm)	Whole width (µm)	Median	*N*	Median
1st	5.04	37.4	455.8	1171.3	0.0224	16^a^	8.5
2nd	3.23	46.2	609.8	1473.8	0.0318	14	8.4
3rd	3.04	48.1	887.3	1555.0	0.0298	15	11.5
4th	4.59	55.1	671.5	1226.3	0.0343	16^a^	7.7
5th	3.64	46.4	651.4	1307.5	0.0297	6	7.3
Median	3.97	47.5	616.3	1397.5	0.0298	67^b^	8.7
Kruskal–Wallis *H*	2015	1.939	9.858	4.805	1.723		6.477
*P*	0.733	0.747	0.043	0.308	0.787		0.166

^a^ While there are only 15 specimens, some defect profiles are markedly “stepped” creating two measurable episodes of developmental stress.

^b^ “swale” defect excluded since not scored for salience.

If we combine the predicted perikymata counts in the occlusal wall from all numbered LEH (Table 6), we can estimate the overall duration of episodes of distress, assuming a RP of 7 days. The median perikymata count from 67 defects (8.7) equates to a duration of 61 days with an interquartile range from 33 to 99 days. Alternatively, we can restrict the analysis to only those LEH in which the occlusal wall perikymata could be directly counted (*n* = 14), which results in a median count of 8.5 perikymata with an inter‐quartile range of 40 to 77 days.

Four of the most cervically‐located episodes of LEH are apparent outliers in that they occurred relatively quickly after their preceding LEH (average interval = 23.3 vs. 54.9, for the other 45 intervals). At an interval of about 5.4 months, these are clearly sub‐annual in their timing. Three of these four LEHs are numbered LEH5. We can ask whether these four late‐occurring LEHs differ metrically from the others (Table 7). While tests of statistical significance cannot be performed on the groups in Table 7 due to small sample size, we think it noteworthy that the last occurring LEH on the crown, from four different males, are deeper, steeper, wider and of longer duration.

**TABLE 7:** Comparison of median descriptive measures of those LEH that appear annually‐spaced vs a minority that are clearly sub‐annual.

LEH spacing	Onset angle (°)	Depth (µm)	Onset width (µm)	Predicted perikymata count in occlusal wall of defect
Circ‐annual (*n* = 64)	3.93	47.5	607.7	8.45
Sub‐annual (*n* = 4)	5.50	87.92	1001.23	12.32

Abbreviation: LEH, linear enamel hypoplasia.

To summarize, our results demonstrate that these 15 male Japanese macaques were experiencing a stress event on average every 12 months lasting about 2 months. We interpret these results to be consistent with a regularly occurring seasonal stressor that likely represents the winter period. In four individual males, there was a final episode of stress, recorded on the dental crown, about 5 months later but of possibly greater severity.

We still need to compare the severity of LEH among species, if possible. We calculated the “enamel deficit ratio” from three thin sections (five measurements per section) where we could count daily increments over a measured distance with a phase contrast microscope (Table 8).

**TABLE 8:** Daily secretion rates (µm); distance between striae of Retzius measured along the enamel prims.

Tooth	Location of measurement (crown/enamel thickness)	Mean of three measurements (µm)	Number of counted increments	Daily secretion rate (µm)
EHUB 10683	Mid‐crown/middle third	37.5	7	5.4
EHUB 10097	Cervical/outer third	43.1	7	6.2
EHUB 10681	Cervical/middle third	32.7	7	4.7
EHUB 10654	Middle/middle third	34.0	7	4.9
EHUB 9828	Apical/middle third	34.9	7	5.0
Mean		36.4	7	5.2

Daily secretion rate for all five canine teeth combined is 5.2 µm. Using this estimate of mean daily secretion rate and a RP of 7 days, the enamel deficit ratio has a median value of 0.17 (interquartile range 0.09–0.32); in other words, enamel from an average LEH furrow is thinned by about 17% of the potential daily enamel secretion.

## 7 Discussion

This is a feasibility study based on male canine teeth from 15 animals, drawn from the northernmost latitudes of the Japanese archipelago, and their developmental experiences may not be representative of monkey populations elsewhere in Japan. Consequently, we consider our results to be indicative, not definitive. Nevertheless, a clear pattern of significant, circ‐annual stress is observed on the teeth. Like Fukuhara (1959), we found a RP of 7 days. Most samples of at least four or more teeth from a population will detect more than one RP (Hogg et al. 2020; Hu, Du, and Zhao 2023), so it is unrealistic to expect an invariant RP in future studies.

That rLEH tends to increase in severity in later forming parts of the canine crown, as we have shown, can be discounted as reflecting, simply enamel geometry (McGrath et al. 2019). Rather, we can conclude that the annually repetitive, presumably winter, developmental stress experienced by juvenile Japanese macaques remains much the same throughout their growth, with the notable exception for four males among whom the last LEH, near the cervix, occurs sooner than expected; probably in the summer months. Male Japanese macaques show a markedly prolonged duration of canine crown formation, up to at least 4 years in some individuals. Earlier (Figure 3) we provided evidence that canine crown formation commences in the second year of life; consequently, assuming an April–June birth (Hamada et al. 1999), the penultimate “winter” LEH in those four affected males probably occurred at age ca. 4.5 years while the last LEH occurred at about age 5 years, in the summer. According to Yamagiwa (2010), average age of male emigration occurs between 4.7 and 5.3 years on the Kinkazan and Yakushima Islands, respectively. Likely, the last LEH on the crowns of these four affected males reflects transfer to a different population, a transition that may involve a period of isolation and social challenges (Sprague 1992); field studies can help to relieve this uncertainty.

The four anomalously short intervals noted above affect the apparent regularity of recurrence of LEH. With them removed, average deviation of recurrence becomes 4.8 perikymata (reduced from 7.2). In other words, if one assumes that LEH recurs annually (and RP = 7 days), the average deviation in recurrent LEH for this population of macaques is 34 days (±9.2%). This estimate of variation of an exogenously‐cued cycle can be compared to the recognized deviation in endogenous circadian rhythms of ±4 h in 24, that is, about 17% (Halberg et al. 1977). Such relatively low deviation of LEH recurrence among Japanese macaques could be interpreted as meaning that successive LEH is caused by the same cycling stressor.

We found that, by combining all LEH events (Table 6), median defect depth is about 47 µm (range 9.8–173.8 µm (*n* = 68). This value may be compared to a much smaller median of 28 µm for defects in great apes in general (but ranging from 15.6 µm in male mountain gorillas (range 6.2–45.8 µm) to 35.6 µm for lowland gorillas (range 10.8–135.1 µm) (McGrath et al. 2018). Only with standardization of instrumentation, increased observer experience and inclusion of a broadened diversity of primates in varying habitats, will we be in a position to judge the significance of reported differences in defect depth. Defect depth also varies with tooth type and enamel geometry, obscuring the meaning of differences in defect depth between populations. Consequently, there is a need to measure relative distress, that is, directly comparable among primates (Skinner 2023) to provide an assessment of the degree to which potential enamel secretion is being reduced. As described above, we found an enamel deficit ratio of 0.17, an estimate of distress that is on the high side compared to *Lufengpithecus lufengensis* (median = 0.12, IQ range 0.08–0.16) and recent *Pongo pygmaeus* (median = 0.10, IQ = 0.06–0.0.16) (the only other species measured using this method) (Skinner and Ji 2024). Such a result, based on a small pilot study, should be considered preliminary. Nevertheless, as a record of seasonal stress among high latitude monkeys, marked LEH with higher enamel deficit ratios, should be expected.

Having established circ‐annual recurrence of developmental stress lasting about two (+/− one) months, we can examine the three potential etiologies for LEH: cold distress, undernutrition, and secondary plant compounds; keeping in mind that the individual animal's own buffering physiology and behavior would likely lessen the interval during which stress might have an impact. Mutsu, Japan has a “warm summer, cold winter” climate modified by marine influence (Meteoblue weather data). Average temperature drops below 0°C for about 55 days, while precipitation is a fairly constant occurrence throughout the year (snow averages about 100 cm per month from December through March (the snowmelt month (Enari and Sakamaki‐Enari 2013)). Coinciding with low temperatures in winter, windspeeds pick up during the winter months adding to potential thermal distress. Significantly, temperature and windspeed show only a single cycle per year. The duration of seasonal cold (ca. 55 days) is consistent with our previously calculated average duration for LEH of 61 days. Exacerbating cold distress, a lean winter season for Japanese macaques on the Shimokita Peninsula is well established (Hamada and Yamamoto 2010). However, as we have seen, only when fat reserves are exhausted should we expect a metabolic challenge sufficiently severe to affect enamel formation. Nakayama, Matsuoka and Watanuki (1999) showed that juveniles who seasonally experienced deep snow conditions fed mainly on dormant winter buds and tree bark, drawing upon fat reserves over an interval lasting about 100 days during which time individual juveniles lost on average about 17% of annually maximum body mass (notably, equivalent to our finding of the degree of enamel thinning). As noted earlier, Shimokita macaques consume mulberry buds and bark for about 8 weeks in the winter (Watanuki et al. 1994); corresponding closely to our estimate of the average duration of LEH in our Shimokita specimens. Clearly, the response to food shortage exposes the young primate to anti‐feedants particularly tannins that, if present in strong enough concentration, impart a bitter astringency which may discourage consumption or actually be toxic (Shimada 2006). Tannins are usually found in large quantities in the bark of trees where they act as a barrier for micro‐organisms and protect the tree (Ashok and Upadhyaya 2012). SPCs, particularly tannins, can affect the growing monogastric mammal by inhibiting protein digestibility leading to weight loss, (Ávila‐Román et al. 2021; Cappai et al. 2014; Lamy et al. 2010; Mehansho, Butler, and Carlson 1987) as well as having deleterious effects on the gastro‐intestinal tract with increased excretion of protein and essential amino acids (Lamy et al. 2016). Towle et al's recent (2024) report of plane‐form enamel defects in monkeys from a more salubrious habitat (Yakushima) than that of the Shimokita macaques, includes figures of canine teeth with repeated bouts of LEH which, given their apparent spacing, could easily be annual and, therefore, indicate that rLEH may not be ascribable to cold or hunger but from exposure to SPCs. Testing such alternatives is becoming more feasible with the recent demonstration that monkey groups on Yakushima can be distinguished by relative differences in concentration of plant secondary metabolites preserved in feces (Tsuchida et al. 2021).

### 7.1 Conclusions

Male canine teeth show four to five circ‐annual enamel defects per crown lasting about 9 weeks, about 1 month less than winter foraging conditions (100 days) on the Shimokita Peninsula. Our preliminary research effort on LEH among high‐latitude Japanese macaques cannot distinguish the relative effects of the triad of cold, hunger and anti‐feedants since they probably co‐occur in the winter months. Certainly, these monkeys have evolved the necessary physiological and behavioral adaptations to deal with seasonal stress. However, as noted earlier, population density of these northernmost macaques is one‐tenth that of those in the south (Hanya 2010). Over geological time, as seasonality increased in the later Neogene and average annual temperatures dropped in East Asia (Jablonski 1998; Li et al. 2020), the primates could adapt, or move south. Migration towards the equator would mitigate the deleterious effects of winter cold and hunger on the infant and juvenile but need not decrease exposure to secondary plant compounds; indeed, less seasonal habitats could exacerbate this potentiality. Consequently, we would not expect the prevalence of LEH, necessarily, to diminish among equatorial primates while, given the added frequency of flowering and fruiting phenophases in the tropics (van Schaik, Terborgh, and Wright 1993), the incidence of LEH could actually increase in extant frugivorous apes (Skinner and Skinner 2017).

## Author Contributions

**Mark Fretson Skinner:** conceptualization (lead), data curation (lead), formal analysis (lead), funding acquisition (supporting), investigation (equal), methodology (lead), project administration (lead), writing–original draft (lead). **Mao Asami:** data curation (equal), formal analysis (equal), investigation (equal), methodology (equal), resources (equal). **Matthew M Skinner:** conceptualization (equal), data curation (equal), formal analysis (supporting), funding acquisition (lead), investigation (supporting), methodology (equal), resources (equal), writing–review & editing (equal). **Akiko Kato:** data curation (equal), formal analysis (equal), investigation (equal), methodology (equal), resources (equal).

## Supporting information

Supplementary information.

Supplementary information.

## Data Availability

Supplementary material to this article, providing images and raw data, can be found online. Raw data are supplied in the Supplemental Data Table S2.

## References

[ajp23713-bib-0001] Agetsuma, N. , and N. Nakagawa . 1998. “Effects of Habitat Differences on Feeding Behaviors of Japanese Monkeys: Comparison Between Yakushima and Kinkazan.” Primates 39, no. 3: 275–289. 10.1007/BF02573077.

[ajp23713-bib-0002] Agrawal, A. A. , and M. G. Weber . 2015. “On the Study of Plant Defence and Herbivory Using Comparative Approaches: How Important Are Secondary Plant Compounds.” Ecology Letters 18, no. 10: 985–991. 10.1641/0006-3568(2001)051[0651:SMBITA]2.0.CO;2. 26248499

[ajp23713-bib-0003] Aoki, K. , S. Mitsutsuka , A. Yamazaki , K. Nagai , A. Tezuka , and Y. Tsuji . 2015. “Effects of Seasonal Changes in Dietary Energy on Body Weight of Captive Japanese Macaques (*Macaca fuscata*): Diet and Body Weight in Captive Macaques.” Zoo Biology 34, no. 3: 255–261. 10.1002/zoo.21210. 25823966

[ajp23713-bib-0004] Ashok, P. K. , and K. Upadhyaya . 2012. “Tannins Are Astringent.” Journal of Pharmacognosy and Phytochemistry 1, no. 3: 45–50.

[ajp23713-bib-0005] Ávila‐Román, J. , J. R. Soliz‐Rueda , F. I. Bravo , et al. 2021. “Phenolic Compounds and Biological Rhythms: Who Takes the Lead?” Trends in Food Science & Technology 113: 77–85. 10.1016/j.tifs.2021.04.050.

[ajp23713-bib-0006] Bowen, W. H. , and G. Koch . 1970. “Determination of Age in Monkeys (*Macaca irus*) on the Basis of Dental Development.” Laboratory Animals 4, no. 1: 113–123. 10.1258/002367770781036481. 5005918

[ajp23713-bib-0007] Bowman, J. E. 1991. Life History, Growth and Development in Young Primates: A Study Using Captive Rhesus Macaques. Cambridge: University of Cambridge.

[ajp23713-bib-0008] Boyde, A. 1970. “The Surface of the Enamel in Human Hypoplastic Teeth.” Archives of Oral Biology 15, no. 9: 897–IN15. 5273410 10.1016/0003-9969(70)90162-7

[ajp23713-bib-0009] Brunet, M. , P. Fronty , M. Sapanet , L. de Bonis , and L. Viriot . 2002. “Enamel Hypoplasia in a Pliocene Hominid From Chad.” Connective Tissue Research 43, no. 2–3: 94–97. 10.1080/03008200290001177. 12489142

[ajp23713-bib-0010] Butler, L. G. 1992. “Antinutritional Effects of Condensed and Hydrolyzable Tannins.” In Plant Polyphenols: Synthesis, Properties, Significance, edited by R. W. Hemingway and P. E. Laks , 693–698. New York: Springer US. 10.1007/978-1-4615-3476-1_40. 1417695

[ajp23713-bib-0011] Cappai, M. G. , P. Wolf , C. Dimauro , W. Pinna , and J. Kamphues . 2014. “The Bilateral Parotidomegaly (Hypertrophy) Induced by Acorn Consumption in Pigs Is Dependent on Individual׳ S Age but not on Intake Duration.” Livestock Science 167: 263–268. 10.1016/j.livsci.2014.06.011.

[ajp23713-bib-0012] Chollet, M. B. , and M. F. Teaford . 2010. “Ecological Stress and Linear Enamel Hypoplasia in *Cebus* .” American Journal of Physical Anthropology 142, no. 1: 1–6. 10.1002/ajpa.21182. 19918987

[ajp23713-bib-0013] Coley, P. D. , and J. A. Barone . 1996. “Herbivory and Plant Defenses in Tropical Forests.” Annual Review of Ecology and Systematics 27, no. 1: 305–335. 10.1146/annurev.ecolsys.27.1.305.

[ajp23713-bib-0014] Dean, M. C. 1987. “Growth Layers and Incremental Markings in Hard Tissues; A Review of the Literature and Some Preliminary Observations About Enamel Structure in *Paranthropus boisei* .” Journal of Human Evolution 16, no. 2: 157–172. 10.1016/0047-2484(87)90074-1.

[ajp23713-bib-0015] Dean, M. C. , and A. E. Scandrett . 1996. “The Relation Between Long‐Period Incremental Markings in Dentine and Daily Cross‐Striations in Enamel in Human Teeth.” Archives of Oral Biology 41: 233–241. 8735009 10.1016/0003-9969(95)00137-9

[ajp23713-bib-0016] Enari, H. 2014. “Snow Tolerance of Japanese Macaques Inhabiting High‐Latitude Mountainous Forests of Japan.” In High Altitude Primates, edited by N. B. Grow , S. Gursky‐Doyen , and A. Krzton , 133–151. New York: Springer. 10.1007/978-1-4614-8175-1_8.

[ajp23713-bib-0017] Enari, H. , and H. Sakamaki . 2010. “Abundance and Morphology of Japanese Mulberry Trees in Response to the Distribution of Japanese Macaques in Snowy Areas.” International Journal of Primatology 31: 904–919.

[ajp23713-bib-0018] Enari, H. , and H. Sakamaki‐enari . 2013. “Influence of Heavy Snow on the Feeding Behavior of Japanese Macaques (*Macaca fuscata*) in Northern Japan.” American Journal of Primatology 75, no. 6: 534–544. 10.1002/ajp.22128. 23436304

[ajp23713-bib-0019] Epple, G. , B. P. Bryant , I. Mezine , and S. Lewis . 2004. “ *Zanthoxylum piperitum* (DC), A Potential Feeding Deterrent for Mammals: Studies With *Microtus ochrogaster* (Wagner).” Pest Management Science 60, no. 7: 624–630. 15260291 10.1002/ps.886

[ajp23713-bib-0020] FitzGerald, C. M. 1998. “Do Enamel Microstructures Have Regular Time Dependency? Conclusions From the Literature and a Large‐Scale Study.” Journal of Human Evolution 35, no. 4: 371–386. 10.1006/jhev.1998.0232. 9774500

[ajp23713-bib-0021] Fooden, J. , and M. Aimi . 2005. “Systematic Review of Japanese Macaques, *Macaca fuscata* (Gray, 1870).” Fieldiana: Zoology 104, no. 104: 1–198.

[ajp23713-bib-0022] Fukuhara, T. 1959. “Comparative Anatomical Studies of the Growth Lines in the Enamel of Mammalian Teeth.” Primates 34, no. 3: 322–332.

[ajp23713-bib-0023] Furuichi, T. , H. Takasaki , and D. S. Sprague . 1982. “Winter Range Utilization of a Japanese Macaque Troop in a Snowy Habitat.” Folia Primatologica 37, no. 1–2: 77–94. 10.1159/000156021 7068055

[ajp23713-bib-0024] Glander, K. E. 1982. “The Impact of Plant Secondary Compounds on Primate Feeding Behavior.” American Journal of Physical Anthropology 25, no. S3: 1–18. 10.1002/ajpa.1330250503.

[ajp23713-bib-0025] Goodman, A. , C. Martinez , and A. Chavez . 1991. “Nutritional Supplementation and the Development of Linear Enamel Hypoplasias in Children From Tezonteopan, Mexico.” The American Journal of Clinical Nutrition 53, no. 3: 773–781. 10.1093/ajcn/53.3.773. 2000834

[ajp23713-bib-0026] Goodman, A. H. , and J. C. Rose . 1990. “Assessment of Systemic Physiological Perturbations From Dental Enamel Hypoplasias and Associated Histological Structures.” American Journal of Physical Anthropology 33: 59–110. 10.1002/ajpa.1330330506.

[ajp23713-bib-0027] Grahn, D. , and H. C. Heller. 2004. The Physiology of Mammalian Temperature Homeostasis. In *Mammalian Thermoregulation* (ITACCS Critical Care Monograph). Cite Seer. https://citeseerx.ist.psu.edu/document?repid=rep1&type=pdf&doi=3ecdb8b42e5dc987cad16c3a3af3cd97557c23b3.

[ajp23713-bib-0028] Guatelli‐Steinberg, D. 2000. “Linear Enamel Hypoplasia in Gibbons (*Hylobates lar carpenteri*).” American Journal of Physical Anthropology 112: 395–410. 10.1002/1096-8644(200007)112:3<395::aid-ajpa9>3.0.co;2-h. 10861355

[ajp23713-bib-0029] Guatelli‐Steinberg, D. , and M. Skinner . 2000. “Prevalence and Etiology of Linear Enamel Hypoplasia in Monkeys and Apes From Asia and Africa.” Folia Primatologica 71: 115–132. 10.1159/000021740. 10828689

[ajp23713-bib-0030] Guatelli‐Steinberg, D. 2003. “Macroscopic and Microscopic Analyses of Linear Enamel Hypoplasia in Plio‐Pleistocene South African Hominins With Respect to Aspects of Enamel Development and Morphology.” American Journal of Physical Anthropology 120: 309–322. 10.1002/ajpa.10148. 12627527

[ajp23713-bib-0031] Guatelli‐Steinberg, D. 2004. “Analysis and Significance of Linear Enamel Hypoplasia in Plio‐Pleistocene Hominins.” American Journal of Physical Anthropology 123: 199–215. 10.1002/ajpa.10324. 14968419

[ajp23713-bib-0032] Guatelli‐Steinberg, D. , and Z. Benderlioglu . 2006. “Brief Communication: Linear Enamel Hypoplasia and the Shift From Irregular to Regular Provisioning in Cayo Santiago Rhesus Monkeys (*Macaca mulatta*).” American Journal of Physical Anthropology 131, no. 3: 416–419. 10.1002/ajpa.20434. 16617431

[ajp23713-bib-0033] Guatelli‐Steinberg, D. , R. J. Ferrell , and J. Spence . 2012. “Linear Enamel Hypoplasia as an Indicator of Physiological Stress in Great Apes: Reviewing the Evidence in Light of Enamel Growth Variation.” American Journal of Physical Anthropology 148, no. 2: 191–204. 10.1002/ajpa.21619. 22610895

[ajp23713-bib-0034] Guatelli‐Steinberg, D. , R. J. Ferrell , J. Spence , T. Talabere , A. Hubbard , and S. Schmidt . 2009. “Sex Differences in Anthropoid Mandibular Canine Lateral Enamel Formation.” American Journal of Physical Anthropology 140, no. 2: 216–233. 10.1002/ajpa.21047. 19350641

[ajp23713-bib-0035] Halberg, F. , F. Carandente , G. Cornelissen , and G. S. Katinas . 1977. “[Glossary of Chronobiology (Author's Transl)].” Chronobiologia 4, no. Suppl 1: 1–189. 352650

[ajp23713-bib-0036] Hamada, Y. , S. Hayakawa , J. Suzuki , and S. Ohkura . 1999. “Adolescent Growth and Development in Japanese Macaques (*Macaca fuscata*): Punctuated Adolescent Growth Spurt By Season.” Primates 40, no. 3: 439–452. 10.1007/BF02557580.

[ajp23713-bib-0037] Hamada, Y. , and A. Yamamoto . 2010. “Chapter 2: Morphological Characteristics, Growth and Aging in Japanese Macaqures.” In The Japanese Macaques, edited by N. Nakagawa , M. Nakamichi , and H. Sugiura , 27–52. New York: Springer. 10.1007/978-4-431-53886-8.

[ajp23713-bib-0038] Hannibal, D. L. , and D. Guatelli‐Steinberg . 2005. “Linear Enamel Hypoplasia in the Great Apes: Analysis by Genus and Locality.” American Journal of Physical Anthropology 127: 13–25. 10.1002/ajpa.20141. 15386281

[ajp23713-bib-0039] Hanya, G. , M. Kiyono , H. Takafumi , R. Tsujino , and N. Agetsuma . 2007. “Mature Leaf Selection of Japanese Macaques: Effects of Availability and Chemical Content.” Journal of Zoology 273, no. 2: 140–147.

[ajp23713-bib-0040] Hanya, G. , M. Kiyono , A. Yamada , et al. 2006. “Not Only Annual Food Abundance but Also Fallback Food Quality Determines the Japanese Macaque Density: Evidence From Seasonal Variations in Home Range Size.” Primates 47, no. 3: 275–278. 10.1007/s10329-005-0176-2. 16432639

[ajp23713-bib-0041] Hanya, G. , N. Ménard , M. Qarro , et al. 2011. “Dietary Adaptations of Temperate Primates: Comparisons of Japanese and Barbary Macaques.” Primates 52: 187–198. 21340696 10.1007/s10329-011-0239-5

[ajp23713-bib-0042] Hanya, G. 2010. “Chapter 4: Ecological Adaptations of Temperate Primates: Population Density of Japanese Macaques.” In The Japanese Macaques, edited by N. Nakagawa , M. Nakamichi , and H. Sugiura , 79–97. New York: Springer.

[ajp23713-bib-0043] Hillson, S. 2014. Tooth Development in Human Evolution and Bioarchaeology. Cambridge: Cambridge University Press. 10.1017/CBO9780511894916.

[ajp23713-bib-0044] Hillson, S. 2017. Enamel hypoplasia = stress? *Stressed Out: Debunking the stress myth in the study of archaeological human remains*. https://www.ucl.ac.uk/archaeology/news-events/conferences-ucl-institute-archaeology.

[ajp23713-bib-0045] Hogg, R. , R. Lacruz , T. G. Bromage , et al. 2020. “A Comprehensive Survey of Retzius Periodicities in Fossil Hominins and Great Apes.” Journal of Human Evolution 149: 102896. 10.1016/j.jhevol.2020.102896. 33069911

[ajp23713-bib-0046] Hori, T. , T. Nakayama , H. Tokura , F. Hara , and M. Suzuki . 1977. “Thermoregulation of the Japanese Macaque Living in a Snowy Mountain Area.” The Japanese Journal of Physiology 27, no. 3: 305–319. 10.2170/jjphysiol.27.305. 410988

[ajp23713-bib-0047] Hu, R. , B. Du , and L. Zhao . 2023. “Retzius Periodicity in the Late Miocene Hominoid *Lufengpithecus lufengensis* From Southwest China: Implications for Dental Development and Life History.” Journal of Human Evolution 181: 103400. 10.1016/j.jhevol.2023.103400. 37307694

[ajp23713-bib-0048] Ishii, Y. , and S. Ohara . 2004. “Quantitative Differences of the Components Between the Inner Bark and the Outer Bark of *Morus alba* .” Journal of the Japanese Forestry Society 86, no. 4: 372–374.

[ajp23713-bib-0049] Ito, M. , and F. Hayashi . 2020. “Tree‐Leaf Chemicals and Feeding Behavior of Arboreal Mammals in Seasonal Environment.” In Co‐Evolution of Secondary Metabolites, edited by J. M. Merillon and K. G. Ramawat , 345–376. New York: Springer.

[ajp23713-bib-0050] Jablonski, N. G. 1998. “The Response of Catarrhine Primates to Pleistocene Environmental Fluctuations in East Asia.” Primates 39, no. 1: 29–37.

[ajp23713-bib-0051] Kawamoto, Y. 2010. “Chapter 3: Modes of Differentiation in Japanese Macaques: Perspectives From Population Genetics.” In The Japanese Macaques, edited by N. Nakagawa , M. Nakamichi , and H. Sugiura , 53–76. New York: Springer.

[ajp23713-bib-0052] Kawamoto, Y. , K.‐i Tomari , S. Kawai , and S. Kawamoto . 2008. “Genetics of the Shimokita Macaque Population Suggest an Ancient Bottleneck.” Primates 49, no. 1: 32–40. 10.1007/s10329-007-0057-y. 17646922

[ajp23713-bib-0053] Kessler, A. , and A. Kalske . 2018. “Plant Secondary Metabolite Diversity and Species Interactions.” Annual Review of Ecology, Evolution, and Systematics 49, no. 1: 115–138. 10.1146/annurev-ecolsys-110617-062406.

[ajp23713-bib-0054] Kierdorf, H. , C. Witzel , E. Bocaege , T. Richter , and U. Kierdorf . 2021. “Assessment of Physiological Disturbances During Pre‐and Early Postnatal Development Based on Microscopic Analysis of Human Deciduous Teeth From the Late Epipaleolithic Site of Shubayqa 1 (Jordan).” American Journal of Physical Anthropology 174, no. 1: 20–34. 33017861 10.1002/ajpa.24156

[ajp23713-bib-0055] Kufeldt, C. , and B. Wood . 2022. “Distinguishing Primate Taxa With Enamel Incremental Variables.” Journal of Human Evolution 164: 103139. 10.1016/j.jhevol.2021.103139. 35123173

[ajp23713-bib-0056] Kumar, R. , and M. Singh . 1984. “Tannins: Their Adverse Role in Ruminant Nutrition.” Journal of Agricultural and Food Chemistry 32, no. 3: 447–453. 10.1021/jf00123a006.

[ajp23713-bib-0057] Kurihara, Y. , K. Kinoshita , I. Shiroishi , and G. Hanya . 2020. “Seasonal Variation in Energy Balance of Wild Japanese Macaques *(Macaca Fucata Yakui*) in a Warm‐Temperate Forest: A Preliminary Assessment in the Coastal Forest of Yakushima.” Primates 61, no. 3: 427–442. 10.1007/s10329-020-00797-3. 32048082

[ajp23713-bib-0058] Lamy, E. , E. S. Baptista , A. V. Coelho , and F. C. Silva . 2010. “Morphological Alterations in Salivary Glands of Mice (*Mus Musculus*) Submitted to Tannin Enriched Diets: Comparison With Sialotrophic Effects of Sympathetic Agonists Stimulation.” Arquivo Brasileiro de Medicina Veterinária e Zootecnia 62: 837–844. 10.1590/S0102-09352010000400012.

[ajp23713-bib-0059] Lamy, E. , C. Pinheiro , L. Rodrigues , et al. (2016). Determinants of Tannin‐rich Food And Beverage Consumption: Oral Perception vs. Psychosocial Aspects. In Tannins: Biochemistry, Food Sources and Nutritional Properties, edited by C. A. Combs , 29–58. New York: Nova Publishers. http://hdl.handle.net/10174/18018.

[ajp23713-bib-0060] Landi, F. , F. Alfieri , I. Towle , A. Profico , and A. Veneziano . 2021. “Fluctuating Asymmetry and Stress in *Macaca fuscata*: Does Captivity Affect Morphology?” Applied Sciences 11, no. 17: 7879. https://www.mdpi.com/2076-3417/11/17/7879.

[ajp23713-bib-0061] Li, P. , C. Zhang , J. Kelley , et al. 2020. “Late Miocene Climate Cooling Contributed to the Disappearance of Hominoids in Yunnan Region, Southwestern China.” Geophysical Research Letters 47, no. 11: e2020GL087741. 10.1029/2020GL087741.

[ajp23713-bib-0062] Lukacs, J. R. 1999. “Interproximal Contact Hypoplasia in Primary Teeth: A New Enamel Defect With Anthropological and Clinical Relevance.” American Journal of Human Biology 11: 718–734. 11533988 10.1002/(SICI)1520-6300(199911/12)11:6<718::AID-AJHB2>3.0.CO;2-7

[ajp23713-bib-0063] Lyaruu, D. M. , M. A. Van Duin , T. J. M. Bervoets , J. H. M. Wöltgens , and A. L. J. J. Bronckers . 1995. “Effects of Vincristine on the Developing Hamster Tooth Germ in Vitro.” Connective Tissue Research 32, no. 1–4: 281–289. 10.3109/03008209509013735. 7554929

[ajp23713-bib-0064] Macho, G. , D. Reid , M. Leakey , N. Jablonski , and A. Beynon . 1996. “Climatic Effects on Dental Development of *Theropithecus oswaldi* From Koobi Fora and Olorgesailie.” Journal of Human Evolution 30, no. 1: 57–70. http://www.sciencedirect.com/science/article/pii/S0047248496900044.

[ajp23713-bib-0065] MacIntosh, A. J. , and M. A. Huffman . 2010. “Topic 3: Toward Understanding the Role of Diet in Host–Parasite Interactions: The Case for Japanese Macaques.” In The Japanese Macaques, 323–344. New York: Springer.

[ajp23713-bib-0066] MacIntosh, A. J. J. , M. A. Huffman , K. Nishiwaki , and T. Miyabe‐Nishiwaki . 2012. “Urological Screening of a Wild Group of Japanese Macaques (*Macaca fuscata yakui*): Investigating Trends in Nutrition and Health.” International Journal of Primatology 33, no. 2: 460–478. 10.1007/s10764-012-9592-5.

[ajp23713-bib-0067] Mahoney, P. , J. J. Miszkiewicz , R. Pitfield , S. H. Schlecht , C. Deter , and D. Guatelli‐Steinberg . 2016. “Biorhythms, Deciduous Enamel Thickness, and Primary Bone Growth: A Test of the Havers‐Halberg Oscillation Hypothesis.” Journal of Anatomy 228: 919–928. 10.1111/joa.12450. 26914945 PMC5341586

[ajp23713-bib-0068] Maruhashi, T. 1980. “Feeding Behavior and Diet of the Japanese Monkey (*Macaca Fuscata Yakui*) on Yakushima Island, Japan.” Primates 21, no. 2: 141–160. 10.1007/BF02374030.

[ajp23713-bib-0069] McFarlane, G. , D. Guatelli‐Steinberg , C. Loch , et al. 2021. “An Inconstant Biorhythm: The Changing Pace of Retzius Periodicity in Human Permanent Teeth.” American Journal of Physical Anthropology 175, no. 1: 172–186. 10.1002/ajpa.24206. 33368148

[ajp23713-bib-0070] McGrath, K. , S. El‐Zaatari , D. Guatelli‐Steinberg , et al. 2018. “Quantifying Linear Enamel Hypoplasia in Virunga Mountain Gorillas and Other Great Apes.” American Journal of Physical Anthropology 166, no. 2: 337–352. 10.1002/ajpa.23436. 29460951

[ajp23713-bib-0071] McGrath, K. , D. Guatelli‐Steinberg , K. Arbenz‐Smith , et al. 2015. “Linear Enamel Hypoplasia Prevalence in Wild Virunga Mountain Gorillas From Rwanda.” American Journal of Physical Anthropology, Annual Meeting Issue Supplement 60: 221.

[ajp23713-bib-0072] McGrath, K. , L. S. Limmer , A.‐L. Lockey , et al. 2021. “3D Enamel Profilometry Reveals Faster Growth but Similar Stress Severity in Neanderthal Versus *Homo Sapiens* Teeth.” Scientific Reports 11, no. 1: 522. 10.1038/s41598-020-80148-w. 33436796 PMC7804262

[ajp23713-bib-0073] McGrath, K. , D. J. Reid , D. Guatelli‐Steinberg , et al. 2019. “Faster Growth Corresponds With Shallower Linear Hypoplastic Defects in Great Ape Canines.” Journal of Human Evolution 137: 102691. 10.1016/j.jhevol.2019.102691. 31704354

[ajp23713-bib-0074] Mehansho, H. , L. G. Butler , and D. M. Carlson . 1987. “Dietary Tannins and Salivary Proline‐Rich Proteins: Interactions, Induction, and Defense Mechanisms.” Annual Review of Nutrition 7, no. 1: 423–440. 10.1146/annurev.nu.07.070187.002231. 3038154

[ajp23713-bib-0075] Miyamoto, A. , K. Minagawa , K. Nohno , et al. 2023. “Prevalence and Cause of Enamel Hypoplasia in Primary Teeth Among 1‐Year‐Old Japanese Children.” The Open Dentistry Journal 17, no. 1. 10.2174/18742106-v17-230303-2022-93.

[ajp23713-bib-0076] Mole, S. , J. A. M. Ross , and P. G. Waterman . 1988. “Light‐Induced Variation in Phenolic Levels in Foliage of Rain‐Forest Plants: I. Chemical Changes.” Journal of Chemical Ecology 14: 1–21. 24276990 10.1007/BF01022527

[ajp23713-bib-0077] Nakagawa, N. 1989. “Bioenergetics of Japanese Monkeys (*Macaca fuscata*) on Kinkazan Island During Winter.” Primates 30, no. 4: 441–460. 10.1007/BF02380873.

[ajp23713-bib-0078] Nakagawa, N. , M. Nakamichi , and H. Sugiura . 2010. The Japanese Macaques. New York: Springer. 10.1007/978-4-431-53886-8.

[ajp23713-bib-0079] Nakayama, T. , T. Hori , T. Nagasaka , H. Tokura , and E. Tadaki . 1971. “Thermal and Metabolic Responses in the Japanese Monkey at Temperatures of 5–38 degrees C.” Journal of Applied Physiology 31, no. 3: 332–337. 10.1152/jappl.1971.31.3.332. 5000068

[ajp23713-bib-0080] Nakayama, Y. , S. Matsuoka , and Y. Watanuki . 1999. “Feeding Rates and Energy Deficits of Juvenile and Adult Japanese Monkeys in a Cool Temperate Area With Snow Coverage.” Ecological Research 14, no. 3: 291–301. 10.1046/j.1440-1703.1999.143306.x.

[ajp23713-bib-0081] Nass, G. G. 1977. “Intra‐Group Variations in the Dental Eruption Sequence of *Macaca fuscata fuscata* .” Folia Primatologica 28, no. 4: 306–314. 10.1159/000155820. 604224

[ajp23713-bib-0082] Nelson, L. (2020). *Hormonal Response to Seasonal Thermal and Ecological Stressors in Japanese Macaques (Macaca fuscata)* Grand Valley State University. https://scholarworks.gvsu.edu/theses/999.

[ajp23713-bib-0083] Newell, E. A. (1998). *Dental Enamel Hypoplasia in Non‐human Primates: A Systematic Assessment of its Occurrence and Distribution* [Doctoral thesis, Temple University].

[ajp23713-bib-0084] O'Hara, M. C. , and D. Guatelli‐Steinberg . 2020. “Differences in Enamel Defect Expression and Enamel Growth Variables in *Macaca Fascicularis* and *Trachypithecus Cristatus* From Sabah, Borneo.” Journal of Archaeological Science 114: 105078. 10.1016/j.jas.2020.105078.

[ajp23713-bib-0085] Okada, M. 1943. “Tissues of Animal Body: Highly Interesting Details of Nippon Studies in Periodic Patterns of Hard Tissues Is Described.” The Shanghai Evening Post 26–30: 32.

[ajp23713-bib-0086] Pflüger, L. S. , K. E. Pink , A. Böck , M. A. Huffman , and B. Wallner . 2019. “On the Sunny Side of (New) Life: Effect of Sunshine Duration on Age at First Reproduction in Japanese Macaques (*Macaca fuscata*).” American Journal of Primatology 81, no. 7: e23019. 10.1002/ajp.23019. 31243793 PMC6773204

[ajp23713-bib-0087] Reid, D. J. , and R. J. Ferrell . 2006. “The Relationship Between Number of Striae of Retzius and Their Periodicity in Imbricational Enamel Formation.” Journal of Human Evolution 50, no. 2: 195–202. 10.1016/j.jhevol.2005.09.002. 16263151

[ajp23713-bib-0088] Sakamaki, H. , H. Enari , T. Aoi , and T. Kunisaki . 2011. “Winter Food Abundance for Japanese Monkeys in Differently Aged Japanese Cedar Plantations in Snowy Regions.” Mammal Study 36, no. 1: 1–10.

[ajp23713-bib-0089] van Schaik, C. P. , J. W. Terborgh , and S. J. Wright . 1993. “The Phenology of Tropical Forests: Adaptive Significance and Consequences for Primary Consumers.” Annual Review of Ecology and Systematics 24: 353–377.

[ajp23713-bib-0090] Schwartz, G. T. , D. J. Reid , and C. Dean . 2001. “Developmental Aspects of Sexual Dimorphism in Hominoid Canines.” International Journal of Primatology 22, no. 5: 837–860. 10.1023/A:1012073601808.

[ajp23713-bib-0091] Sha, J. C. M. , Y. Kurihara , Y. Tsuji , et al. 2018. “Seasonal Variation of Energy Expenditure in Japanese Macaques (*Macaca fuscata)* .” Journal of Thermal Biology 76: 139–146. 10.1016/j.jtherbio.2018.07.009. 30143288

[ajp23713-bib-0092] Shimada, T. 2006. “Salivary Proteins as a Defense Against Dietary Tannins.” Journal of Chemical Ecology 32: 1149–1163. 10.1007/s10886-006-9077-0. 16770710

[ajp23713-bib-0093] Skinner, M. F. 1986. “Enamel Hypoplasia in Sympatric Chimpanzee and Gorilla.” Human Evolution 1: 289–312. 10.1007/bf02436704.

[ajp23713-bib-0094] Skinner, M. F. 2021. “Cold Discomfort: A Model to Explain Repetitive Linear Enamel Hypoplasia Among *Pan troglodytes* and *Pan paniscus* .” International Journal of Primatology 42, no. 3: 370–403. 10.1007/s10764-021-00206-6.

[ajp23713-bib-0095] Skinner, M. F. 2023. “Meaningful Measures of Enamel Hypoplasia: Prevalence and Comparative Intensity of Developmental Stress.” American Journal of Biological Anthropology 180, no. 4: 761–767. 10.1002/ajpa.24699. 36790765

[ajp23713-bib-0096] Skinner, M. F. , L. K. Delezene , M. M. Skinner , and P. Mahoney . 2024. “Linear Enamel Hypoplasia in *Homo Naledi* Reappraised in Light of New Retzius Periodicities.” American Journal of Biological Anthropology 184, no. 3: e24927. 10.1002/ajpa.24927. 38433613

[ajp23713-bib-0097] Skinner, M. F. , T. L. Dupras , and S. Moya‐Sola . 1995. “Periodicity of Linear Enamel Hypolasia Among Miocene *Dryopithecus* From Spain.” Journal of Paleopathology 7: 195–222.

[ajp23713-bib-0098] Skinner, M. F. , and D. Hopwood . 2004. “Hypothesis for the Causes and Periodicity of Repetitive Linear Enamel Hypoplasia in Large, Wild African (Pan Troglodytesandgorilla Gorilla) and Asian (*Pongo pygmaeus*) Apes Hypothesis for the Causes and Periodicity of Repetitive Linear Enamel Hypoplasia (Rleh) in Large, Wild African (*Pan troglodytes* and *Gorilla gorilla*) and Asian (*Pongo pygmaeus*) Apes.” American Journal of Physical Anthropology 123: 216–235. 10.1002/ajpa.10314. 14968420

[ajp23713-bib-0099] Skinner, M. F. , and X. Ji . 2024. “Detecting the Presence of Different Retzius Periodicities at the Population Level From Repetitive Linear Enamel Hypoplasia Among Lufengpithecus Lufengensis and *Pongo pygmaeus* .” American Journal of Biological Anthropology: e25014. 10.1002/ajpa.25014. 39508616

[ajp23713-bib-0100] Skinner, M. F. , and J. D. Pruetz . 2012. “Reconstruction of Periodicity of Repetitive Linear Enamel Hypoplasia From Perikymata Counts on Imbricational Enamel Among Dry‐Adapted Chimpanzees (*Pan troglodytes* verus) From Fongoli, Senegal.” American Journal of Physical Anthropology 149: 468–482. 10.1002/ajpa.22145. 23041791

[ajp23713-bib-0101] Skinner, M. F. , and M. M. Skinner . 2017. “Orangutans, Enamel Defects, and Developmental Health: A Comparison of Borneo and Sumatra.” American Journal of Primatology 79, no. 8: e22668. 10.1002/ajp.22668. 28407267

[ajp23713-bib-0102] Sprague, D. S. 1992. “Life History and Male Intertroop Mobility Among Japanese Macaques (*Macaca fuscata*).” International Journal of Primatology 13: 437–454.

[ajp23713-bib-0103] Sugiyama, Y. , and H. Ohsawa . 1982. “Population Dynamics of Japanese Monkeys With Special Reference to the Effect of Artificial Feeding.” Folia Primatologica 39, no. 3–4: 238–263. 10.1159/000156080. 7166288

[ajp23713-bib-0104] Swindler, D. R. , and D. Meekins . 1991. “Dental Development of the Permanent Mandibular Teeth in the Baboon, *Papio Cynocephalus* .” American Journal of Human Biology 3, no. 6: 571–580. 10.1002/ajhb.1310030606. 28524287

[ajp23713-bib-0105] Takeshita, R. S. C. , F. B. Bercovitch , M. A. Huffman , et al. 2014. “Environmental, Biological, and Social Factors Influencing Fecal Adrenal Steroid Concentrations in Female Japanese Macaques (*Macaca fuscata*).” American Journal of Primatology 76, no. 11: 1084–1093. 10.1002/ajp.22295. 24839268

[ajp23713-bib-0106] Takeshita, R. S. C. , F. B. Bercovitch , K. Kinoshita , and M. A. Huffman . 2018. “Beneficial Effect of Hot Spring Bathing on Stress Levels in Japanese Macaques.” Primates 59, no. 3: 215–225. 10.1007/s10329-018-0655-x. 29616368

[ajp23713-bib-0107] Thompson, C. L. , B. L. Powell , S. H. Williams , G. Hanya , K. E. Glander , and C. J. Vinyard . 2017. “Thyroid Hormone Fluctuations Indicate a Thermoregulatory Function in Both a Tropical (*Alouatta Palliata*) and Seasonally Cold‐Habitat (*Macaca fuscata*) Primate.” American Journal of Primatology 79, no. 11: e22714. 10.1002/ajp.22714. 29048740

[ajp23713-bib-0108] Towle, I. , and J. D. Irish . 2019. “A Probable Genetic Origin for Pitting Enamel Hypoplasia on the Molars of Paranthropus Robustus.” Journal of Human Evolution 129: 54–61. 10.1016/j.jhevol.2019.01.002. 30904040

[ajp23713-bib-0109] Towle, I. , C. Loch , M. Martínez de Pinillos , M. Modesto‐Mata , and L. J. Hlusko . 2024. “Severe Enamel Defects in Wild Japanese Macaques.” International Journal of Zoology 2024: 8445492. 10.1155/2024/8445492.

[ajp23713-bib-0110] Trotter, M. , B. B. Hixon , and B. J. MacDonald . 1977. “Development and Size of the Teeth of *Macaca mulatta* .” American Journal of Anatomy 150, no. 1: 109–127. 10.1002/aja.1001500108. 412407

[ajp23713-bib-0111] Tsuchida, S. , T. Hattori , A. Sawada , K. Ogata , J. Watanabe , and K. Ushida . 2021. “Fecal by LC‐MS/MS and LC‐QTOF‐MS.” Journal of Veterinary Medical Science 83, no. 6: 1012–1015. 33952783 10.1292/jvms.21-0076 PMC8267200

[ajp23713-bib-0112] Tsuji, Y. , T. Y. Ito , K. Wada , and K. Watanabe . 2015. “Spatial Patterns in the Diet of the Japanese Macaque *Macaca fuscata* and Their Environmental Determinants.” Mammal Review 45, no. 4: 227–238. 10.1111/mam.12045.

[ajp23713-bib-0113] Tsuji, Y. , N. Kazahari , M. Kitahara , and S. Takatsuki . 2008. “A More Detailed Seasonal Division of the Energy Balance and the Protein Balance of Japanese Macaques (*Macaca fuscata*) on Kinkazan Island, Northern Japan.” Primates 49, no. 2: 157–160. 10.1007/s10329-007-0070-1. 18026899

[ajp23713-bib-0114] Tsuji, Y. 2010. “Chapter 5: Regional, Temporal, and Interindividual Variation in the Feeding Ecology of Japanese Macaques.” In The Japanese Macaques, edited by N. Nakagawa , M. Nakamichi , and H. Sugiura , 99–127. New York: Springer.

[ajp23713-bib-0115] Tsuji, Y. , and S. Takatsuki . 2004. “Food Habits and Home Range Use of Japanese Macaques on an Island Inhabited By Deer.” Ecological Research 19: 381–388.

[ajp23713-bib-0116] Tuyen, P. , T. Xuan , D. Khang , et al. 2017. “Phenolic Compositions and Antioxidant Properties in Bark, Flower, Inner Skin, Kernel and Leaf Extracts of *Castanea crenata* Sieb. et Zucc.” Antioxidants 6, no. 2: 31. 28475126 10.3390/antiox6020031 PMC5488011

[ajp23713-bib-0117] Ueno, M. , and M. Nakamichi . 2016. “Japanese Macaque (*Macaca fuscata*) Mothers Huddle With Their Young Offspring Instead of Adult Females for Thermoregulation.” Behavioural Processes 129: 41–43. 10.1016/j.beproc.2016.05.008. 27262980

[ajp23713-bib-0118] Watanuki, Y. , Y. Nakayama , S. Azuma , and S. Ashizawa . 1994. “Foraging on Buds and Bark of Mulberry Trees by Japanese Monkeys and their Range Utilization.” Primates 35, no. 1: 15–24. 10.1007/BF02381482.

[ajp23713-bib-0119] Wrangham, R. W. , and P. G. Waterman . 1983. “Condensed Tannins in Fruits Eaten by Chimpanzees.” Biotropica 15, no. 3: 217–222. http://www.jstor.org/stable/2387832.

[ajp23713-bib-0120] Yamagiwa, J. 2010. “Chapter 1: Research History of Japanese Macaques in Japan.” In The Japanese Macaques, edited by N. Nakagawa , M. Nakamichi , and H. Sugiura , 3–25. New York: Springer. 10.1007/978-4-431-53886-8.

[ajp23713-bib-0121] Zhang, P. , K. Watanabe , and T. Eishi . 2007. “Habitual Hot‐Spring Bathing by a Group of Japanese Macaques (*Macaca fuscata*) in Their Natural Habitat.” American Journal of Primatology 69, no. 12: 1425–1430. 10.1002/ajp.20454. 17554750

