# Supplementary material for: Potential of Japanese Macaques for Understanding Etiology and Seasonality of Repetitive Linear Enamel Hypoplasia in Nonhuman Primates
Source: Am J Primatol. 2024 Dec 17;87(1):e23713. doi: 10.1002/ajp.23713 (PMC11653062; doi:10.1002/ajp.23713)
Supplement: Supplementary file 1 — Supplementary information. [file AJP-87-e23713-s001.pdf]

Japanese macaques
EHUB canines, SEMs, coated casts, thin sections

9822 SEM248

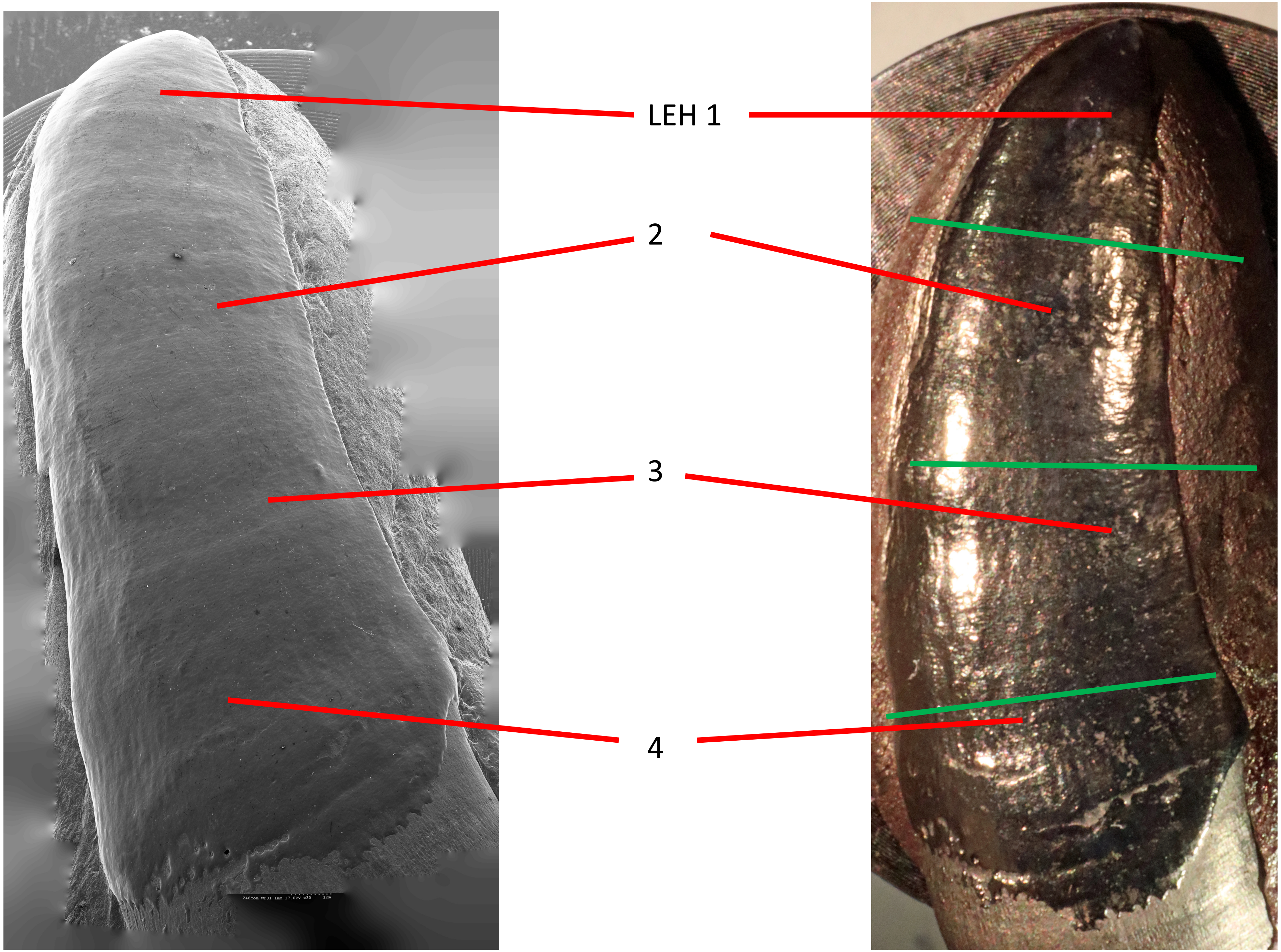

9823 SEM249

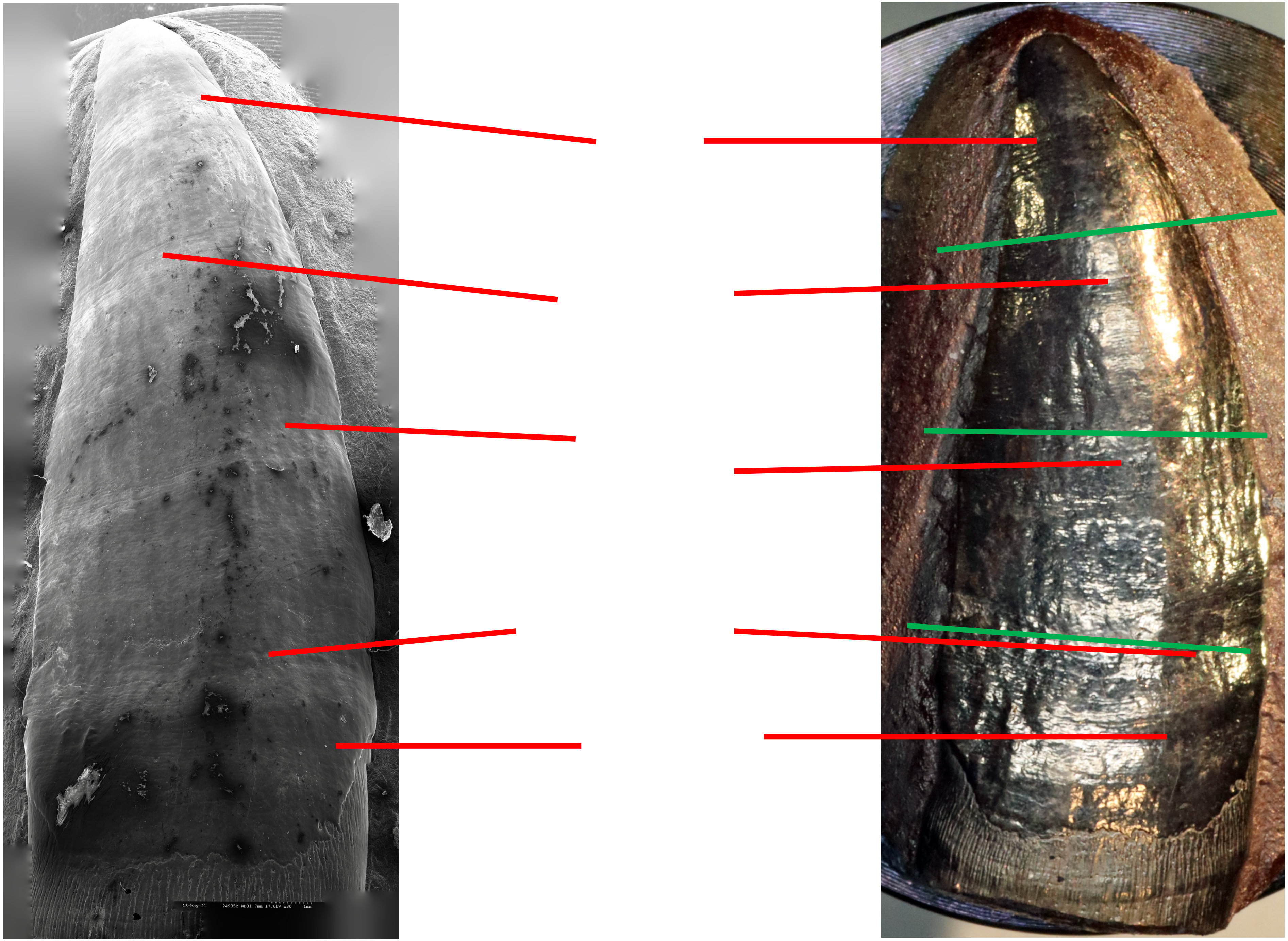

9828 SEM250

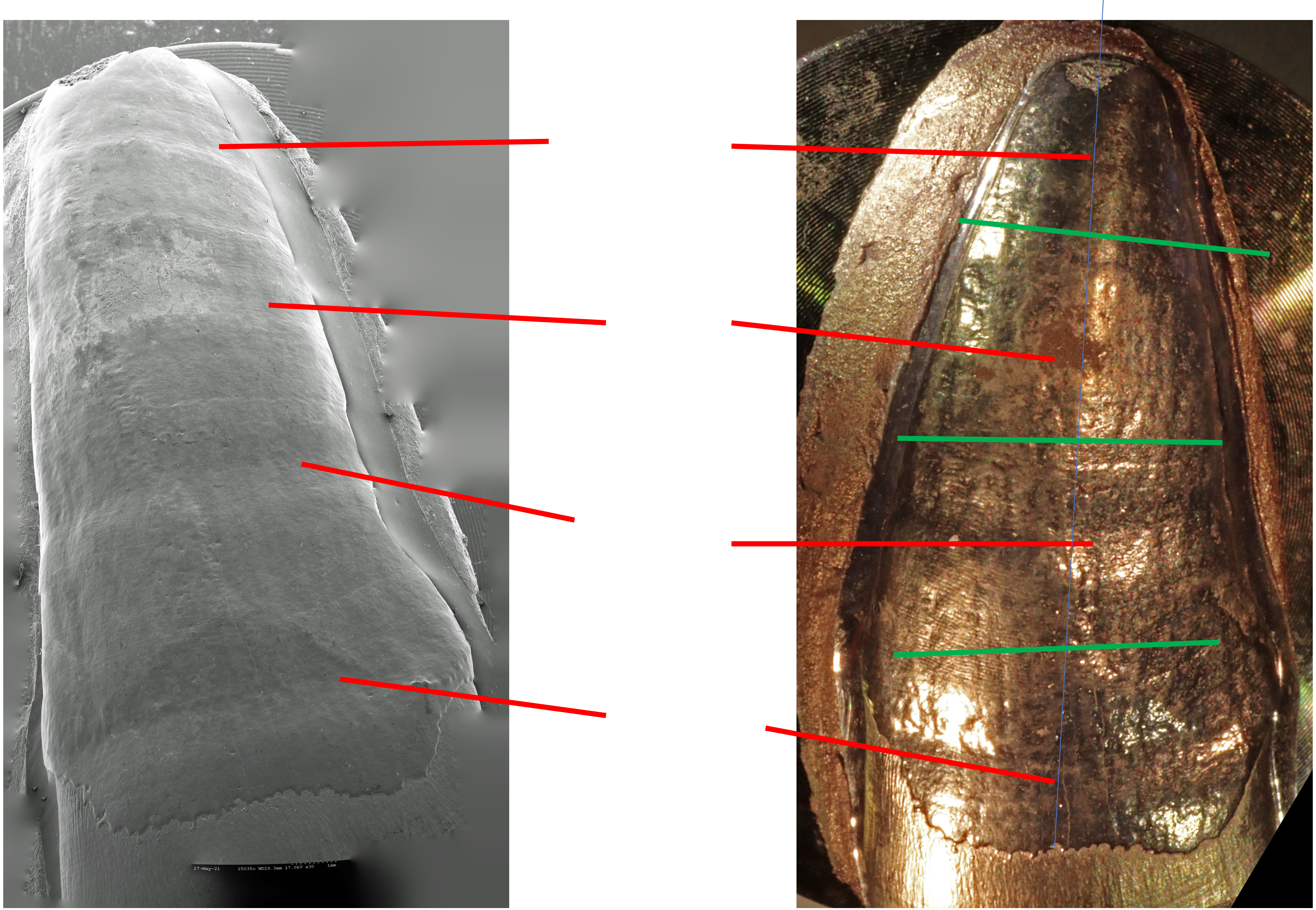

9858 SEM252

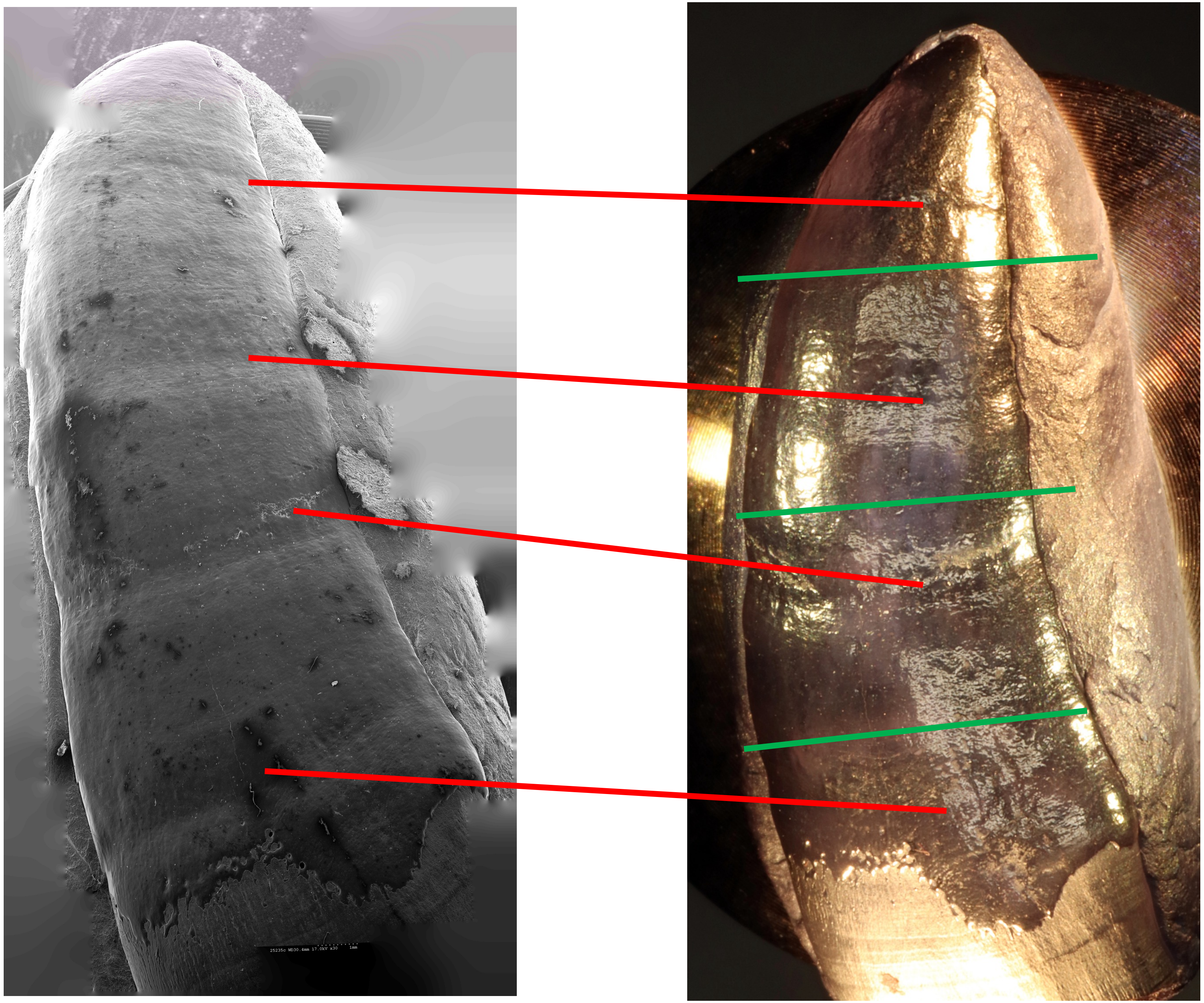

9861 SEM253

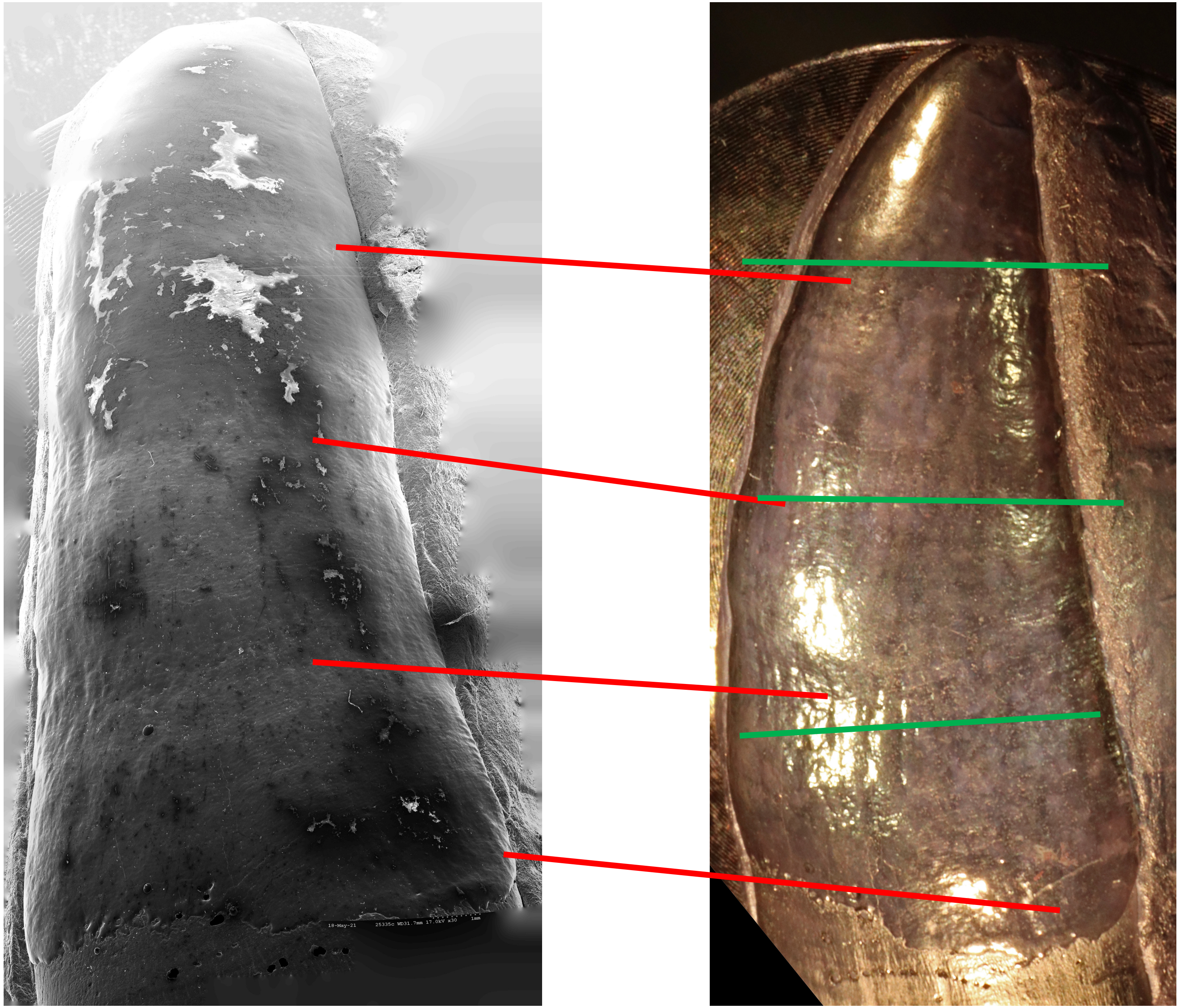

10074 SEM254

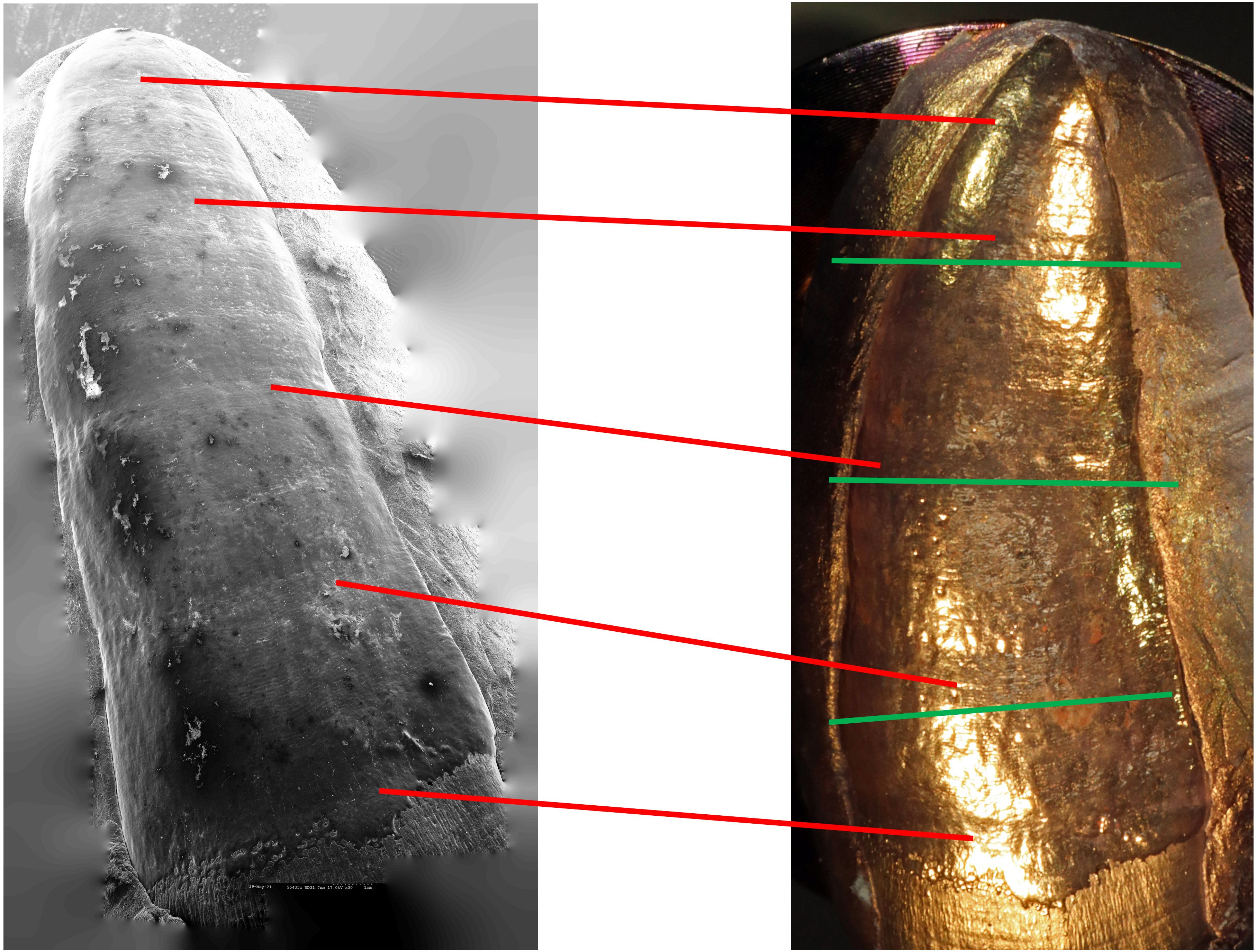

10077 SEM256

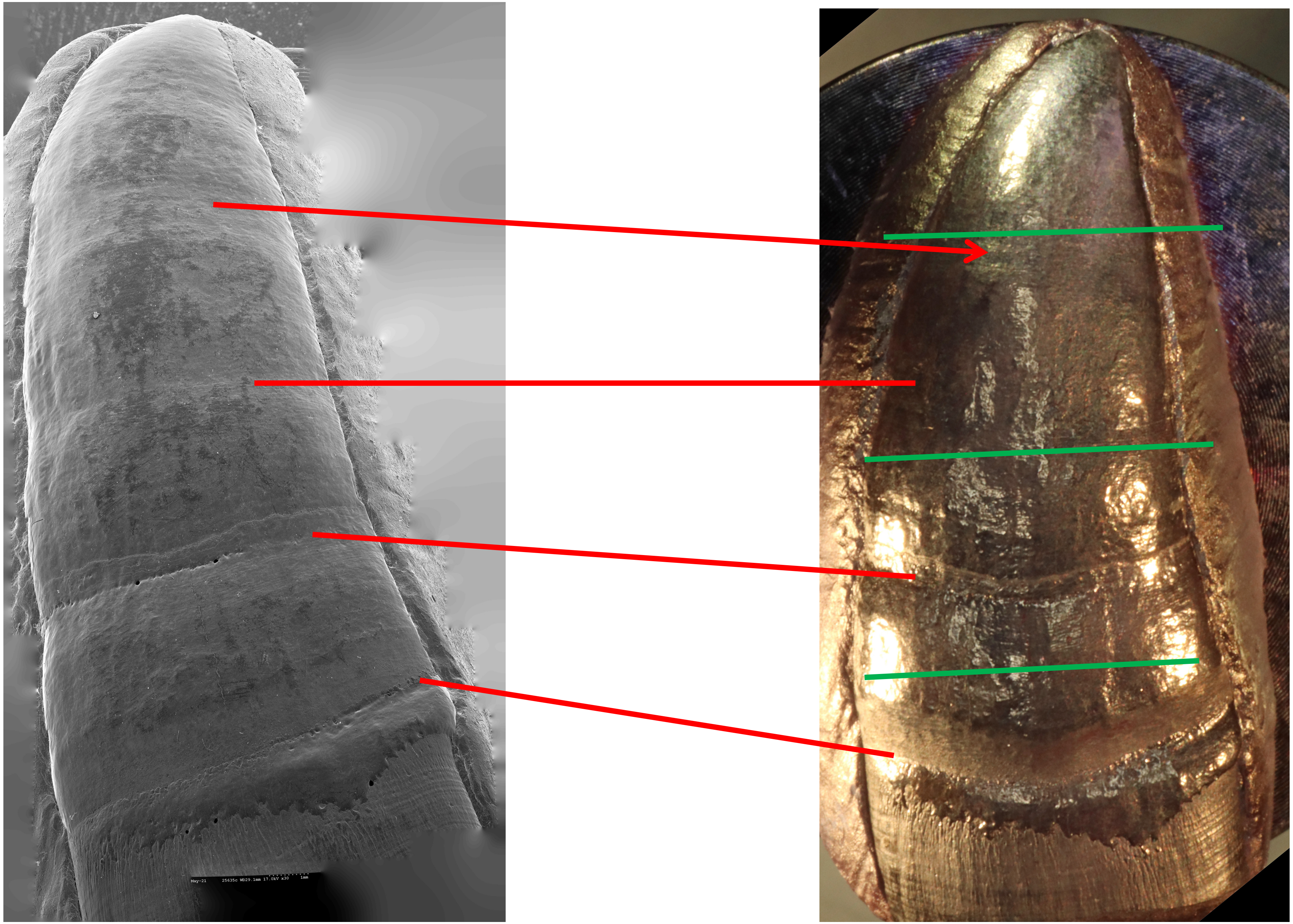

10081 SEM257

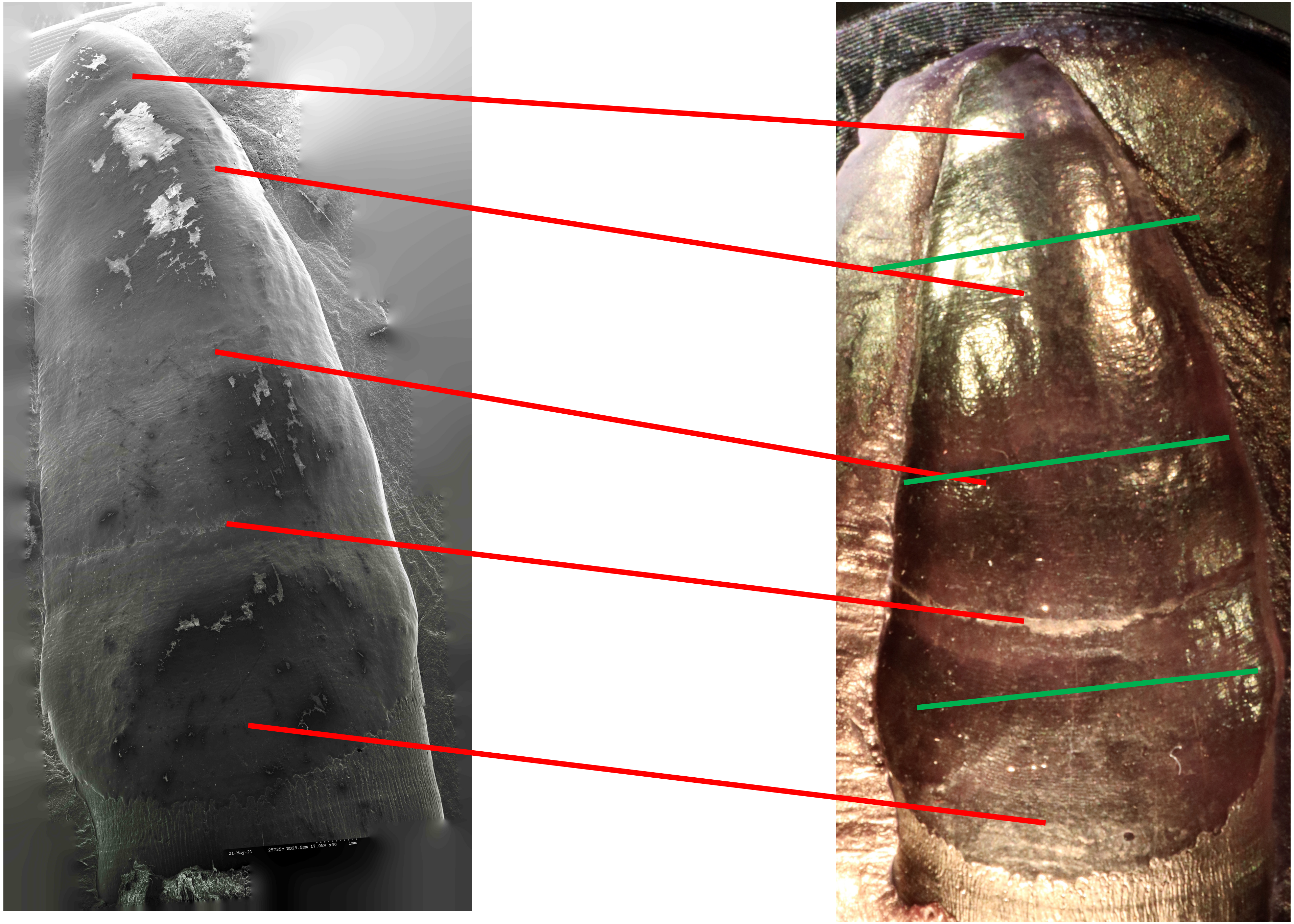

10088 SEM258

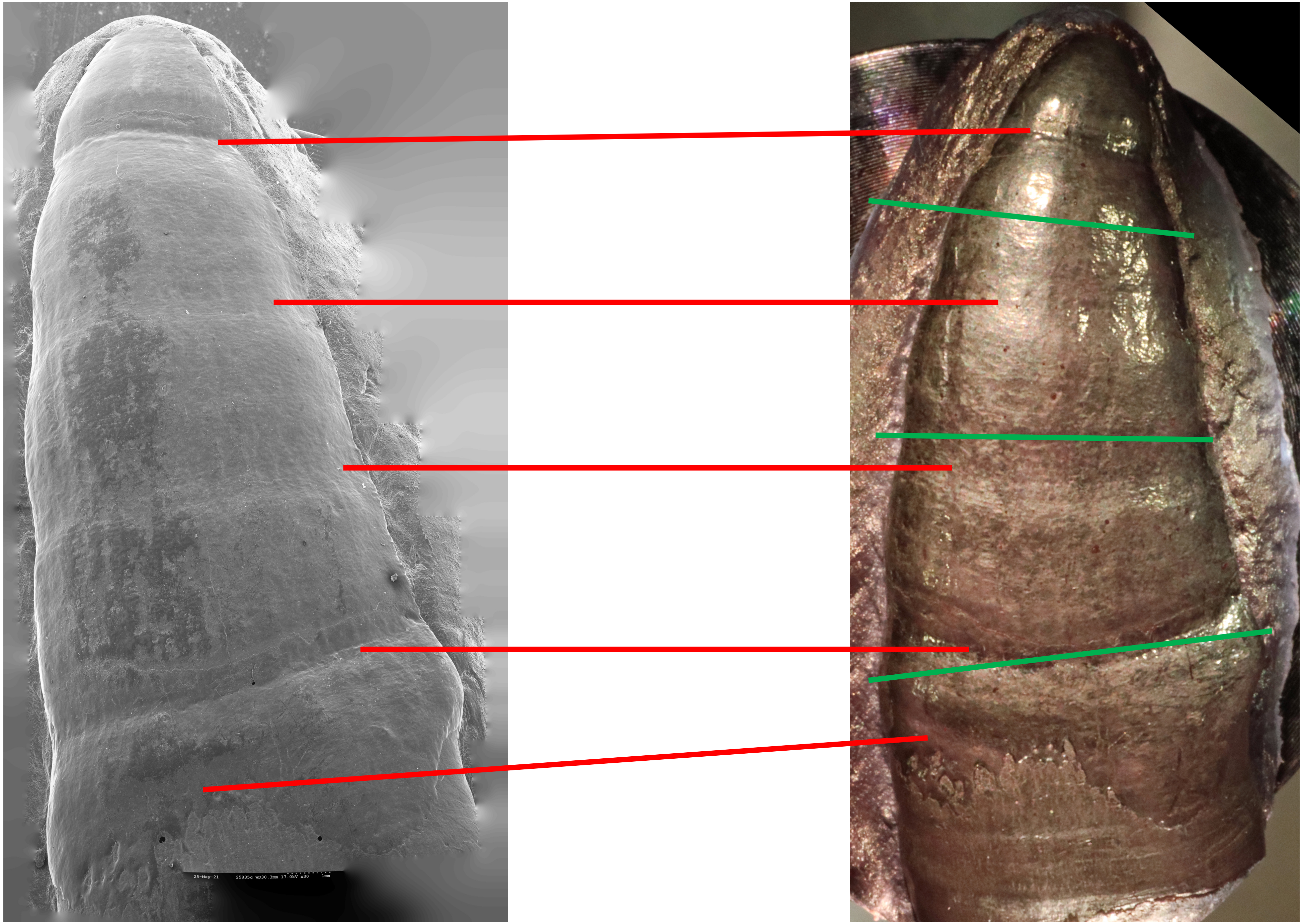

10092 SEM259

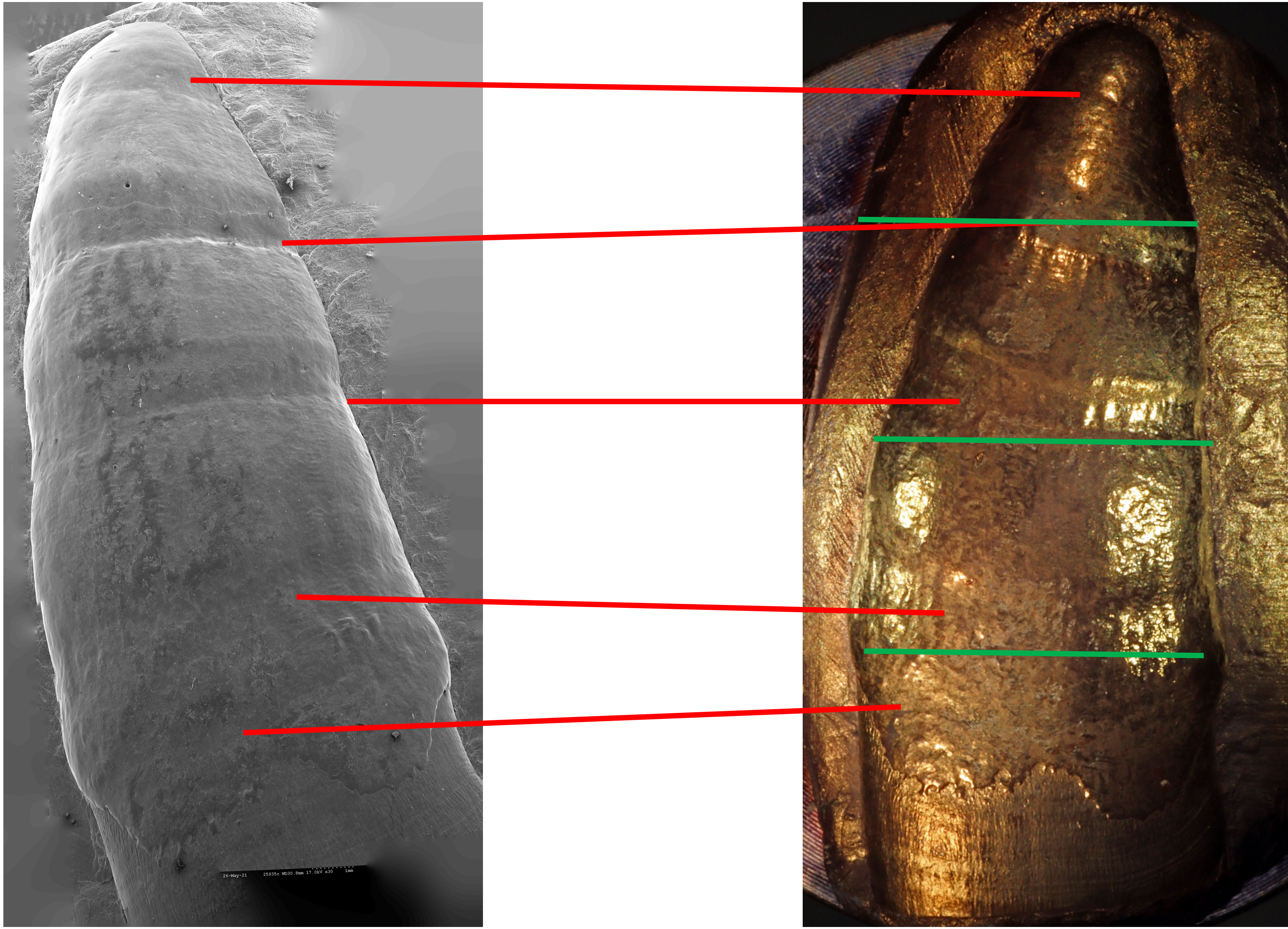

10097 SEM260

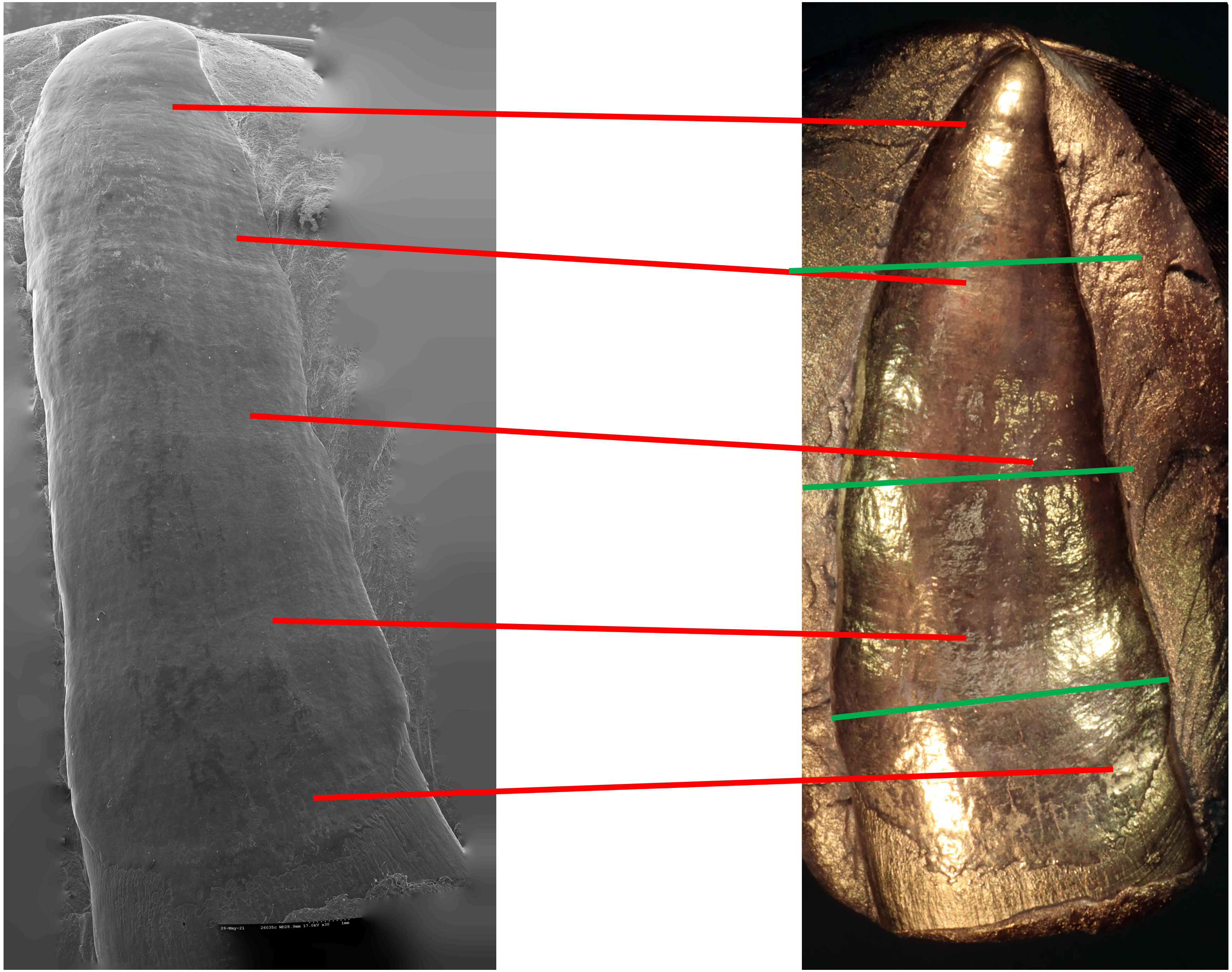

10654 SEM265

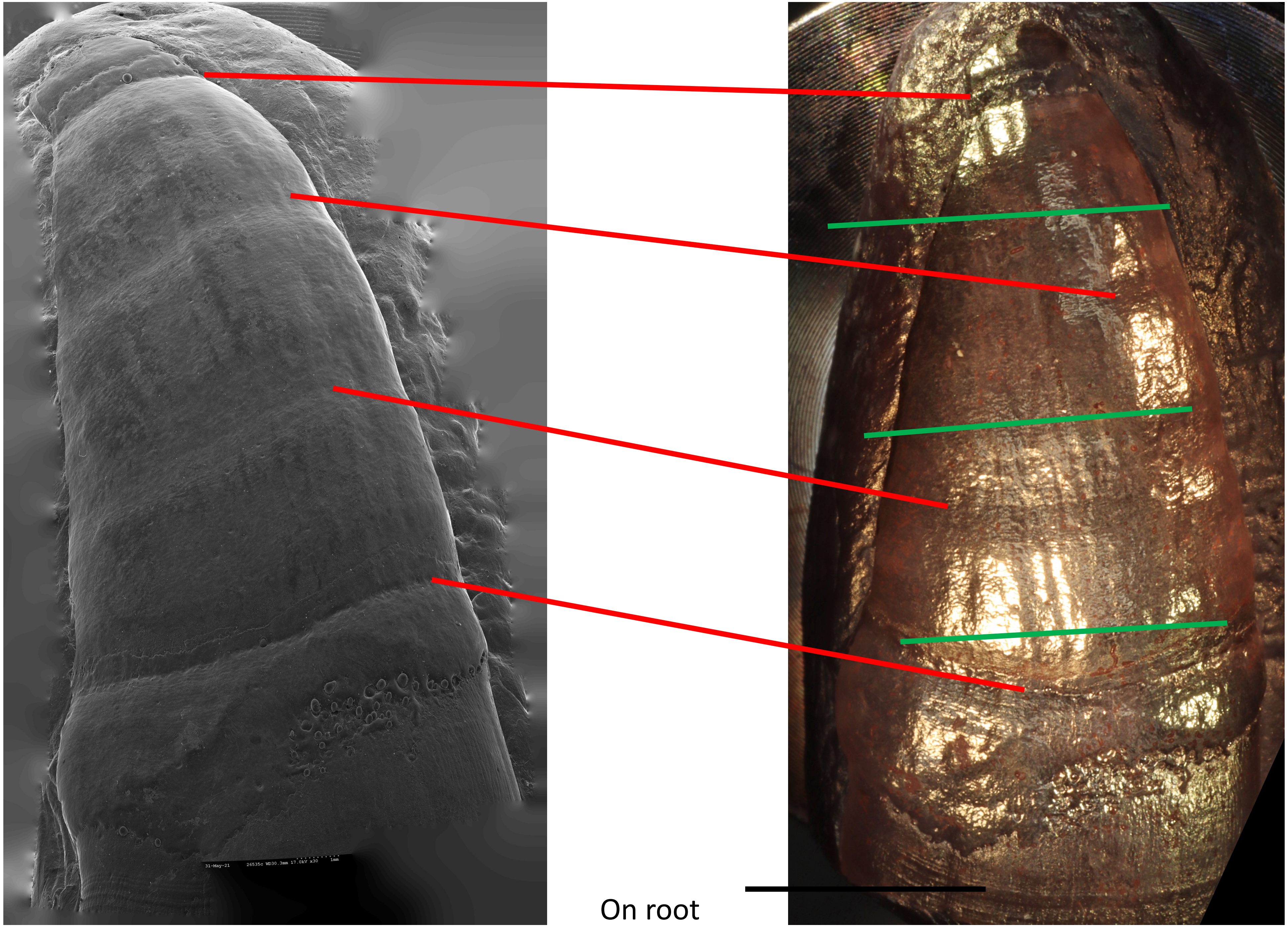

10655 SEM266

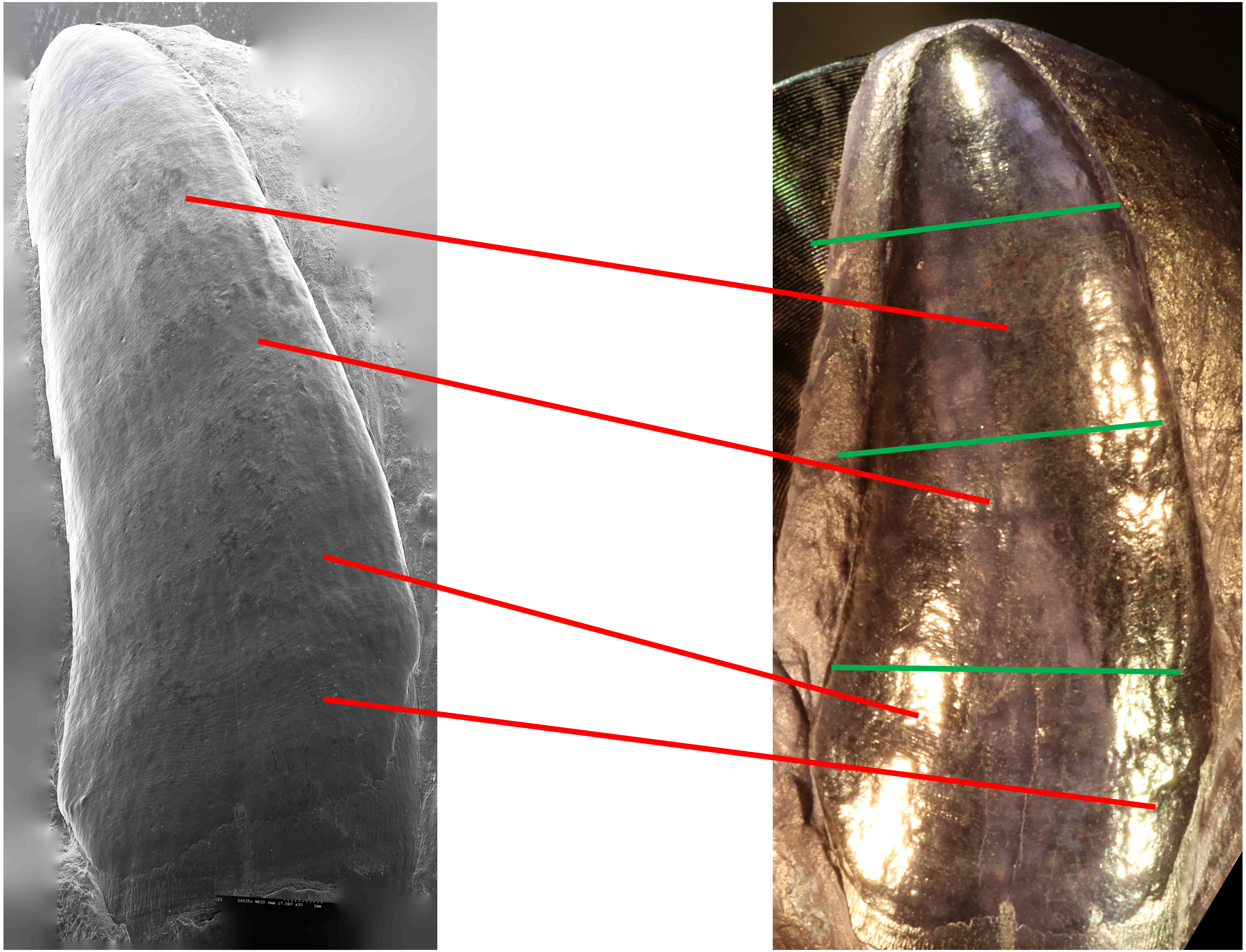

10681 SEM267

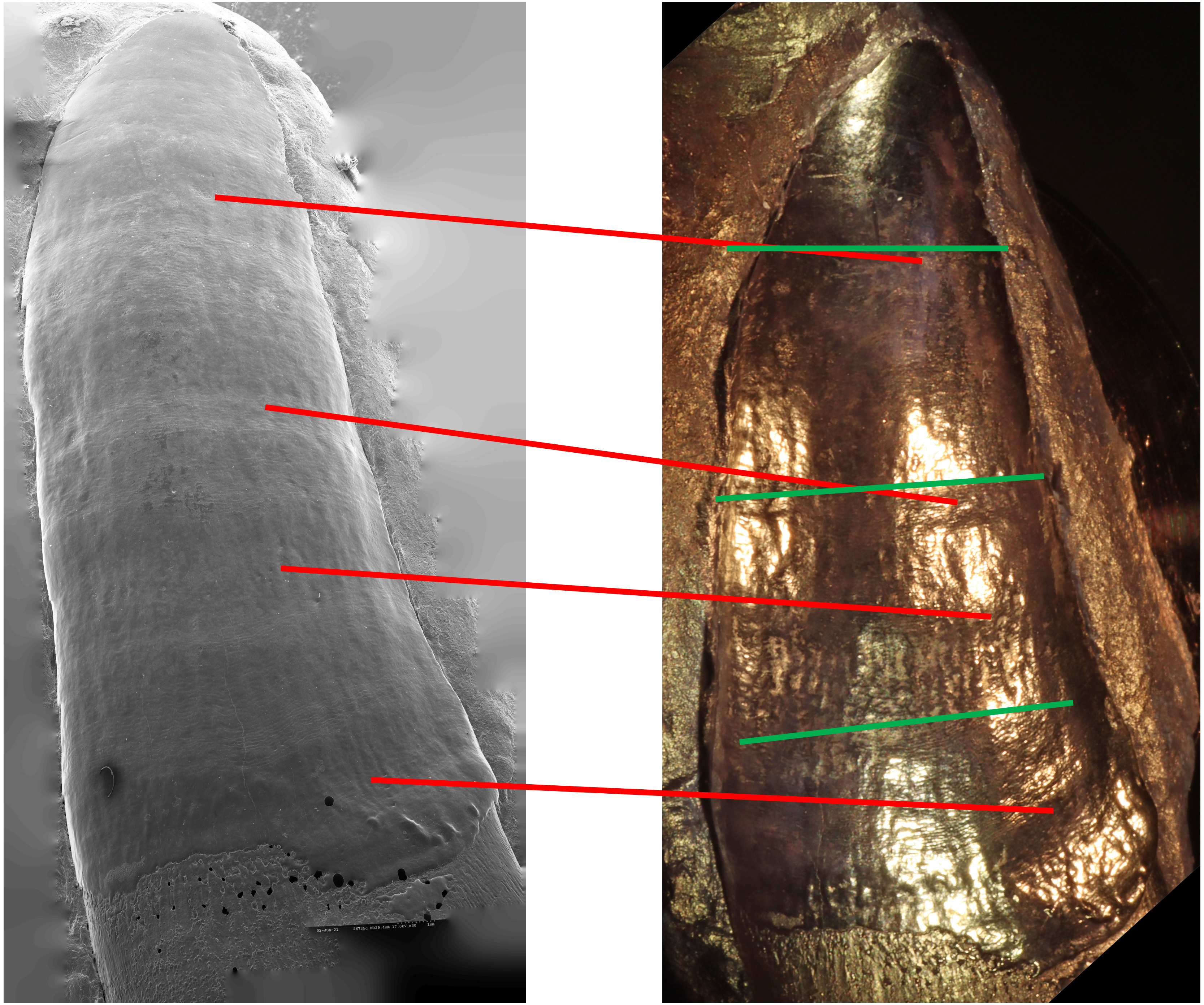

10683 SEM268

LEH 3 is very wide furrow swale

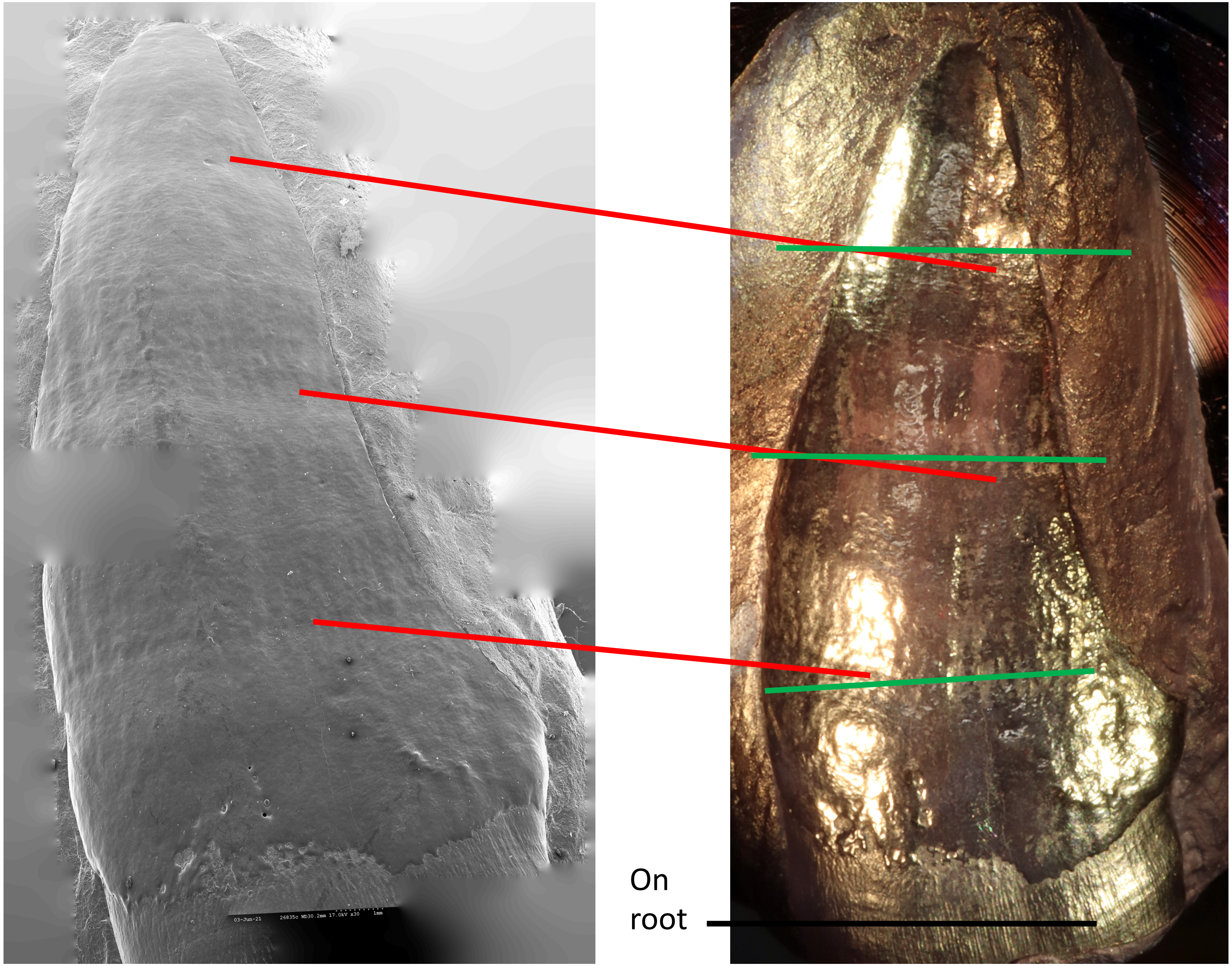

On root

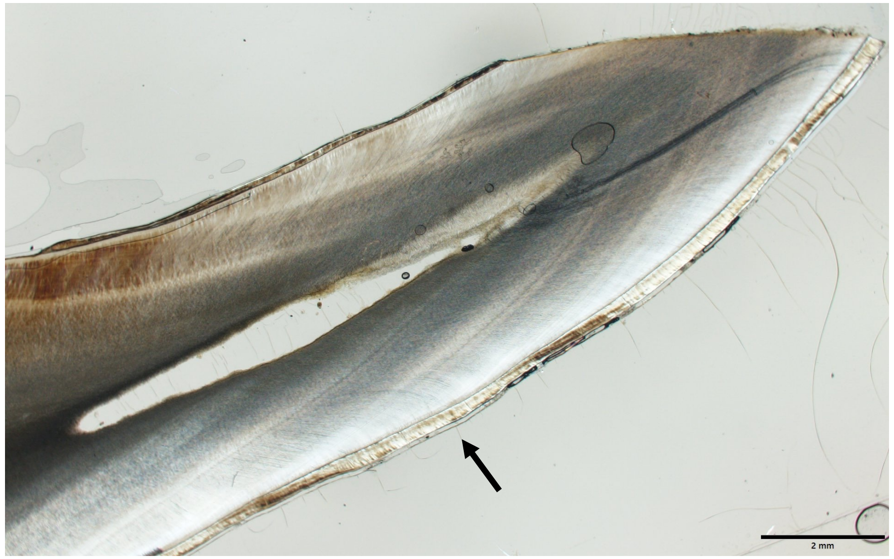

X1.25

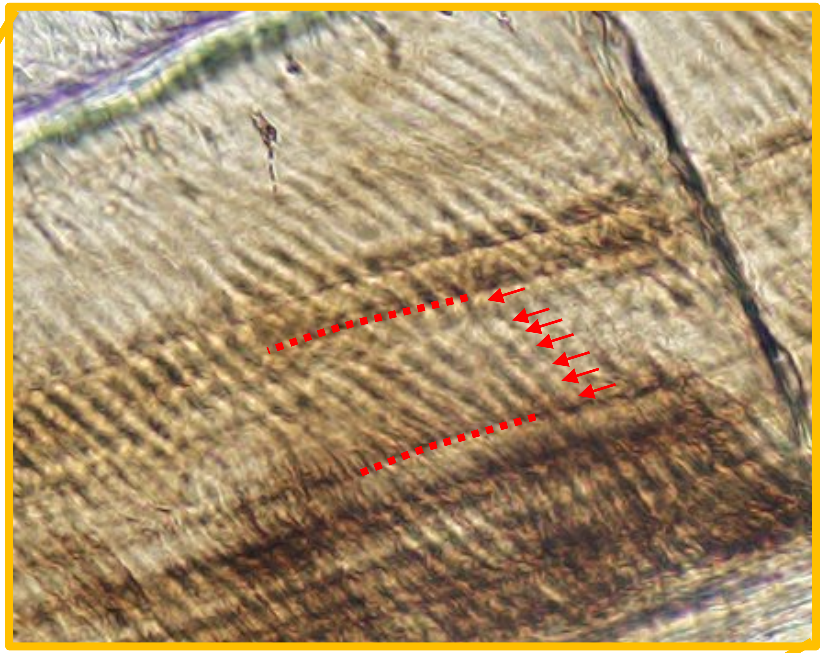

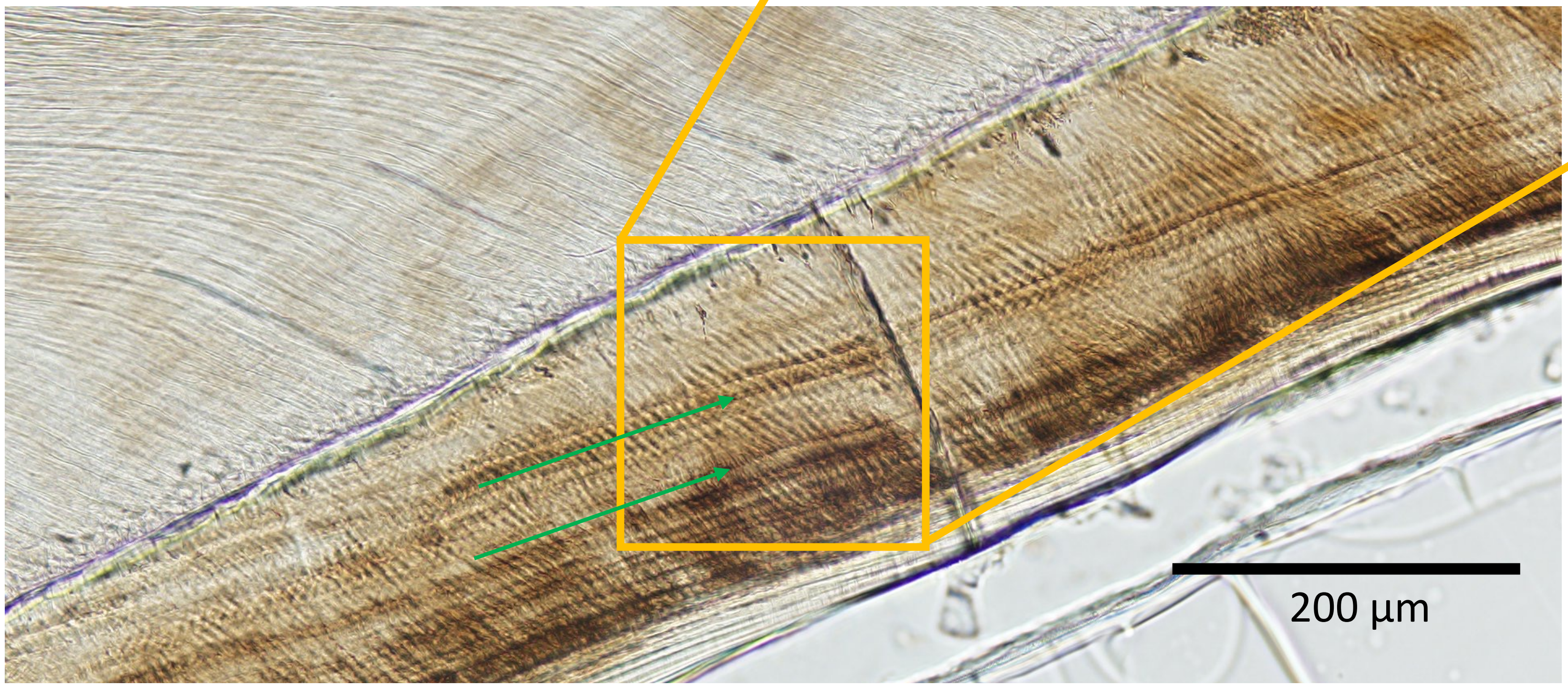

EHUB 10683 (URC) X 200

EHUB 10097 (URC) X 400

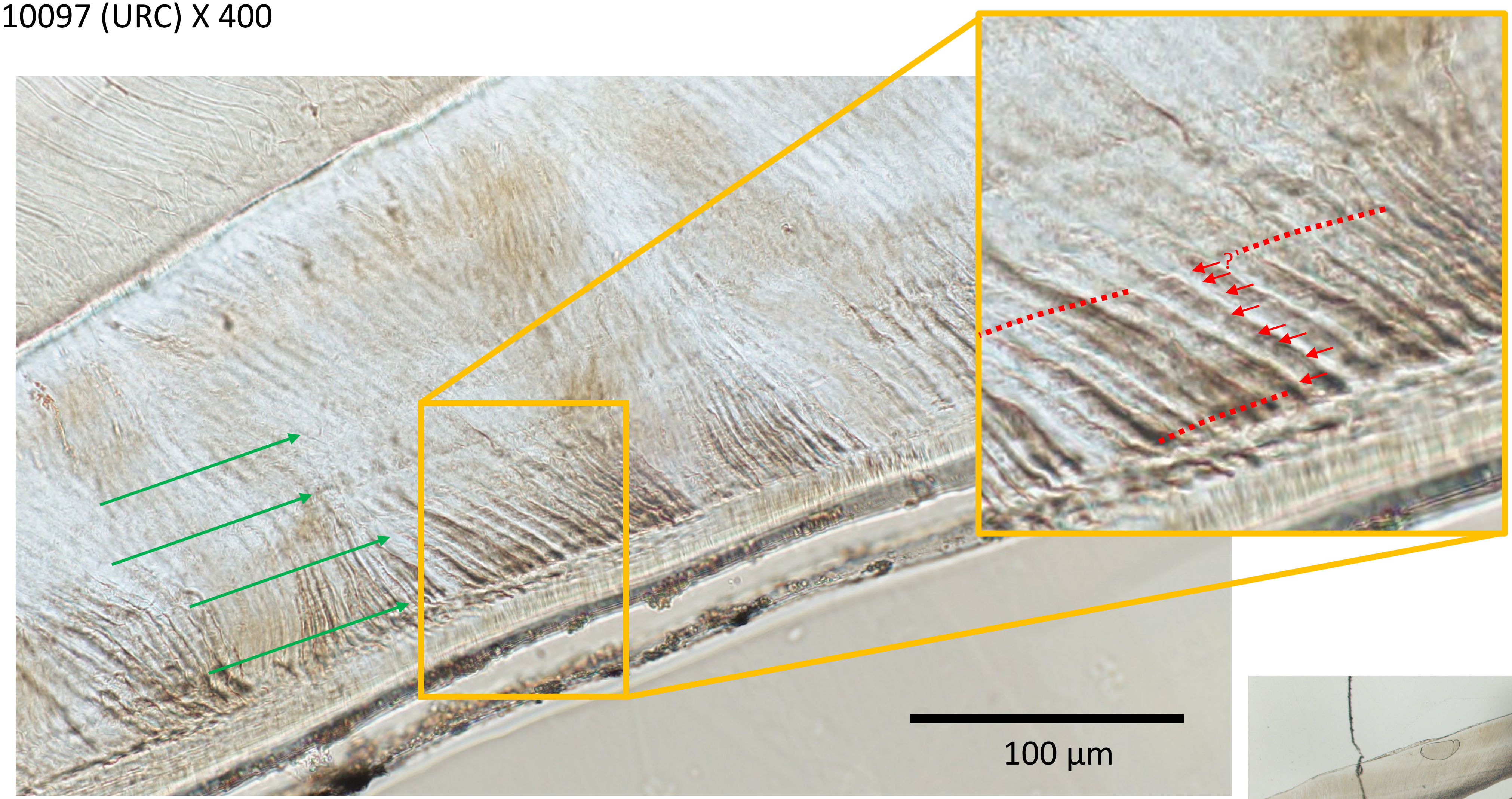

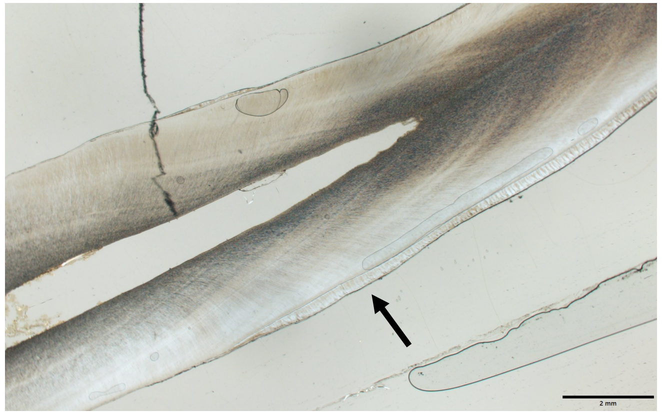

X1.25

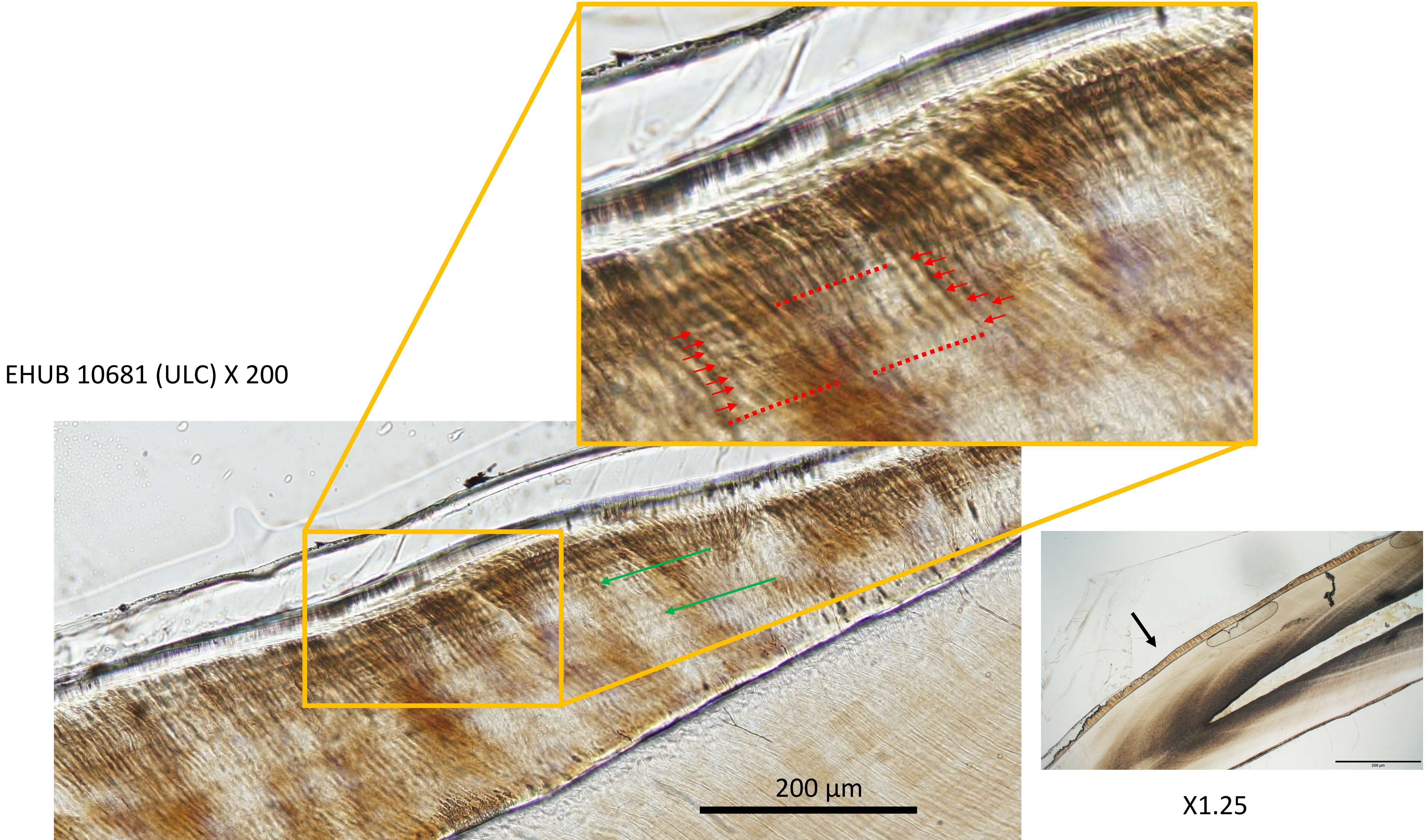
EHUB 10681 (ULC) X 200
200 µm
X1.25

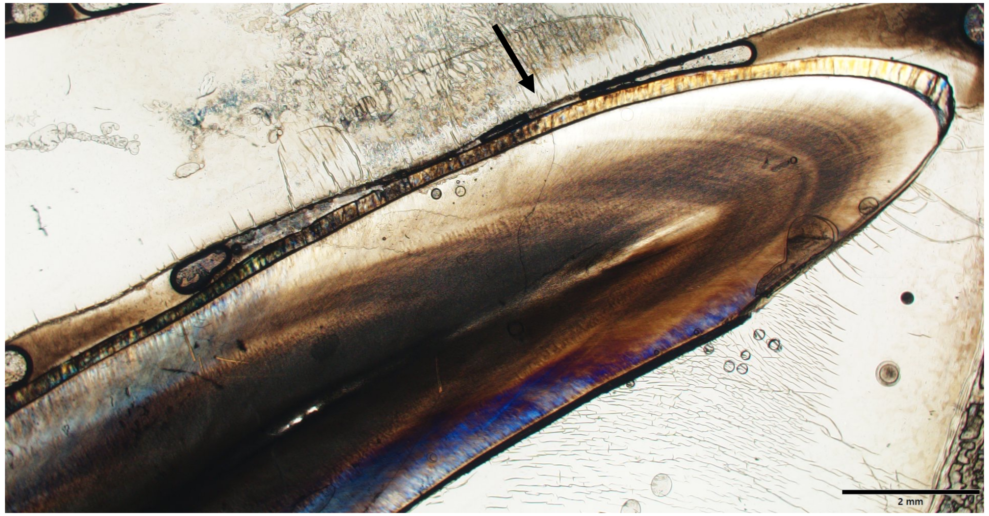

X1.25

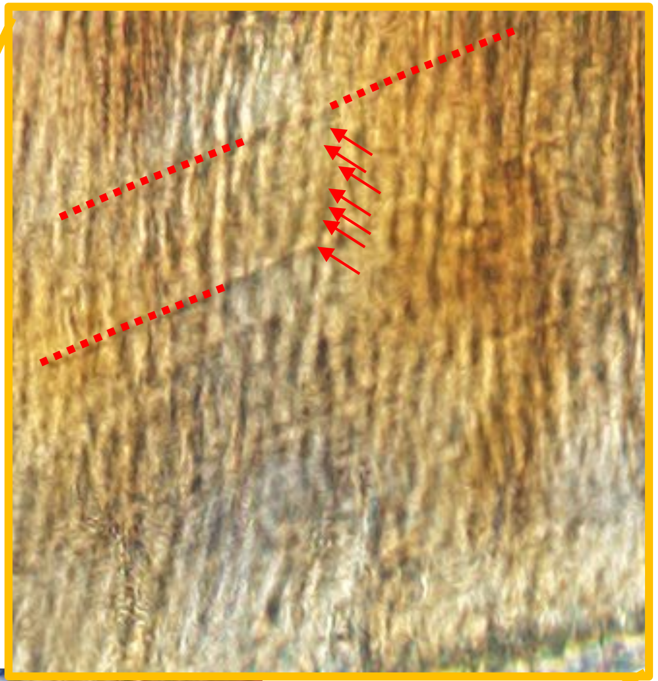

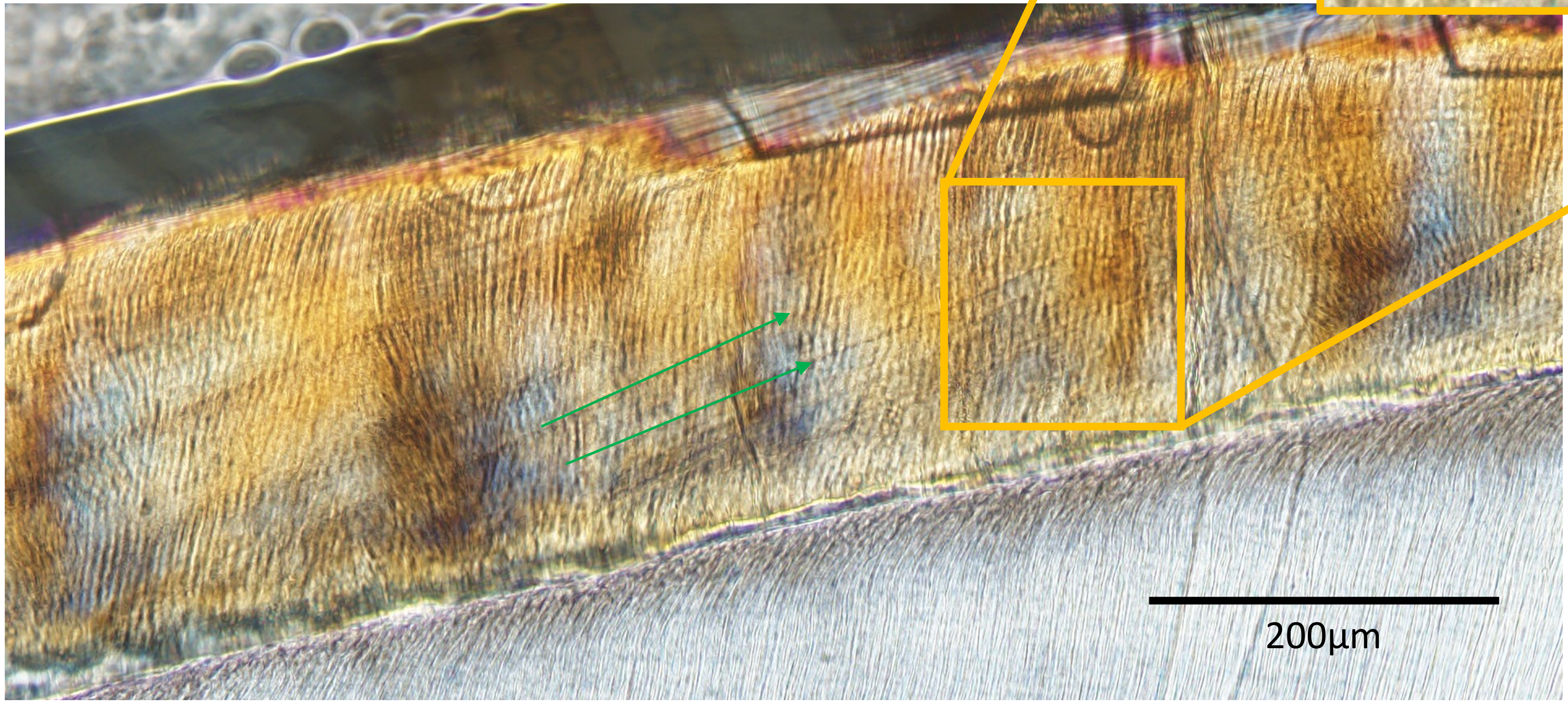

EHUB 9828 (URC) X 200
