# Supplementary material for: Potential of Japanese Macaques for Understanding Etiology and Seasonality of Repetitive Linear Enamel Hypoplasia in Nonhuman Primates
Source: Am J Primatol. 2024 Dec 17;87(1):e23713. doi: 10.1002/ajp.23713 (PMC11653062; doi:10.1002/ajp.23713)
Supplement: Supplementary file 2 — Supplementary information. [file AJP-87-e23713-s002.pdf]

Individual ID	LEH numbered	Location of LEH by crown quarter	Crown quarter for majority of LEH interval	Depth (microns)	Width (microns)	Onset angle (degrees)	Total LEH width (microns)	Calculated median Pk width per crown quarter (microns)
9822	1	1	1	168.0	498.0	18.64	765.0	52.9
	2	2	2	64.6	63.1	41.63	1210.0	62.8
	3	3	3	68.1	1099.5	3.12	2040.0	75.3
	4	4		54.9	1350.5	2.33	2202.5	89.3
9823	1	1	1	31.7	438.9	0.65	1397.5	52.9
	2	2	2	9.8	238.1	2.35	371.2	62.8
	3	3	3	21.4	806.0	1.52	1022.5	75.3
	4	4	4	38.9	476.6	4.67	887.5	89.3
	5	4		52.6	178.3	14.34	925.0	89.3
9828	1	1	2	17.4	194.3	2.69	652.5	52.9
	2	2	3	35.1	539.7	3.18	1590.0	62.8
	3	3	4	117.0	814.7	8.03	2122.5	75.3
	4	4		86.1	683.9	4.16	2182.5	89.3
9858	1	1	2	33.6	392.0	4.90	542.5	52.9
	2	2	2	122.8	964.7	7.25	1725.0	62.8
	3	3	3	106.7	1418.6	4.30	1555.0	75.3
	4	4		60.4	331.5	10.33	955.0	89.3
9861	1	2	2	33.3	557.1	2.67	1372.5	62.8
	2	3	3	19.8	200.0	5.65	1957.5	75.3
	3	3	4	91.6	811.1	6.44	1392.5	75.3
	4	4		64.9	238.1	2.67	685.0	89.3
10074	1	1	1	23.3	191.2	1.79	510.0	52.9
	2	1	2	68.2	159.2	20.27	1250.0	52.9
	3	2	3	25.3	137.4	10.31	437.5	62.8
	4	3	4	28.4	659.2	2.47	1065.0	75.3
	5	4		64.1	1474.6	2.49	1812.5	89.3
10077	1	2	2	33.6	1128.9	1.70	1355.0	62.8
	2	2	3	55.6	191.1	11.59	1132.5	62.8
	3	3	4	58.7	277.8	11.94	437.5	75.3
	4	4		94.0	688.7	7.78	1205.0	89.3
10081	1	1	1	53.2	547.3	5.32	1700.0	52.9
	2	2	2	11.2	765.0	0.84	1765.0	62.8
	3	3	3	27.3	867.4	1.52	1407.5	75.3
	4	3	4	55.3	402.5	4.52	1247.5	75.3
	5	4		34.0	439.7	3.90	1082.5	89.3
10088	1	1	1	108.3	787.1	7.60	1442.5	52.9
	2	2	2	47.4	616.3	2.50	1357.5	62.8
	3	3	3	31.9	1476.4	1.24	1600.0	75.3

	4	3	4	171.0	1062.5	9.14	2510.0	75.3
	5	4		35.3	422.1	4.06	950.0	89.3
10092	1	1	1	12.5	611.9	1.17	747.5	52.9
	2	2	2	110.9	603.4	3.27	1875.0	62.8
	3	2	3	62.6	1194.5	3.00	1717.5	62.8
	4	3	4	136.9	1232.9	6.34	1782.5	75.3
	5	4		40.2	863.1	2.67	1747.5	89.3
10097	1	1	1	48.2	461.9	5.19	1557.5	52.9
	2	2	2	45.0	1006.2	2.56	1630.0	62.8
	3	2	3	48.1	887.3	2.90	1722.5	62.8
	4	3	4	47.5	1207.3	2.25	1842.5	75.3
	5	4		78.6	1329.5	3.38	1532.5	89.3
10654	1	1	1	51.9	166.7	13.24	395.0	52.9
	1	1		119.0	432.2	9.24	1670.0	52.9
	2	2	2	84.5	1219.1	3.97	1960.0	62.8
	3	3	3	27.9	1380.2	1.16	1920.0	75.3
	4	4		35.7	466.8	4.38	612.5	89.3
	4	4		27.4	47.9	29.77	152.5	89.3
	4	4		27.3	139.1	8.93	470.0	89.3
10655	1	2	2	41.1	206.2	9.31	812.5	62.8
	2	3	3	26.6	624.9	2.29	1132.5	75.3
	3	4	4	31.2	940.0	1.83	1500.0	89.3
	4	4		43.8	688.5	3.64	1555.0	89.3
10681	1	2	2	17.1	449.7	1.02	987.5	62.8
	2	3	3	22.9	625.2	2.10	1347.5	75.3
	3	3	3	113.6	899.4	7.20	1537.5	75.3
	4	4		84.9	741.8	6.53	1572.8	89.3
10683	1	2	2	173.8	874.2	8.06	2067.5	62.8
	2	3	3	34.1	253.9	5.38	1212.5	75.3
	3	3		30.4	572.6	3.04	1960.0	75.3

Interval	Salience	Median PK width per crown quarter	Corrected LEH interval (mm) on SEM	Predicted PK for LEH interval allowing for Inter-furrow crown quarter	PK per onset width using LEXT relevant crown quarter	New DSR where known for tooth or 5.2 micron mean	Enamel decrement ratio when RP is 7	Total area missing enamel (mm2)	Observed PK in occlusal wall
One to Two	marked	52.9	3.31	63	9.4	5.20	0.49	0.0643	7
Two to Three	mild	62.8	3.31	53	1.0	5.20	1.76	0.0391	
Three to Four	mild	75.3	3.04	40	14.6	5.20	0.13	0.0695	
	mild				15.1	5.20	0.10	0.0605	
One to Two	mild	52.9	2.99	57	8.3	5.20	0.10	0.0221	
Two to Three	mild	62.8	4.38	70	3.8	5.20	0.07	0.0018	
Three to Four	mild	75.3	4.18	55	10.7	5.20	0.06	0.0110	
Four to Five	marked	89.3	1.88	21	5.3	5.20	0.20	0.0173	
	mild				2.0	5.20	0.72	0.0243	
One to Two	mild	62.8	2.92	47	3.7	5.00	0.14	0.0057	4
Two to Three	mild	75.3	2.99	40	8.6	5.00	0.12	0.0279	11
Three to Four	mild	89.3	4.10	46	10.8	5.00	0.31	0.1241	
	mild				7.7	5.00	0.32	0.0940	
One to Two	mild	62.8	3.75	60	7.4	5.20	0.12	0.0091	6
Two to Three	mild	62.8	3.75	60	15.4	5.20	0.22	0.1059	7
Three to Four	marked	75.3	4.14	55	18.8	5.20	0.16	0.0830	
	mild				3.7	5.20	0.45	0.0288	
One to Two	mild	62.8	3.50	56	8.9	5.20	0.10	0.0229	10
Two to Three	mild	75.3	3.87	51	2.7	5.20	0.20	0.0194	
Three to Four	mild	89.3	4.21	47	10.8	5.20	0.23	0.0638	11
	faint				2.7	5.20	0.67	0.0222	
One to Two	mild	52.9	3.92	74	3.6	5.20	0.18	0.0059	
Two to Three	mild	62.8	4.57	73	3.0	5.20	0.62	0.0426	
Three to Four	mild	75.3	4.35	58	2.2	5.20	0.32	0.0055	
Four to Five	mild	89.3	5.05	57	8.8	5.20	0.09	0.0151	
	mild				16.5	5.20	0.11	0.0581	
One to Two	mild	62.8	3.65	58	18.0	5.20	0.05	0.0227	
Two to Three	mild	75.3	3.52	47	3.0	5.20	0.50	0.0315	
Three to Four	marked	89.3	3.81	43	3.7	5.20	0.44	0.0128	
	marked				7.7	5.20	0.33	0.0567	16
One to Two	marked	52.9	2.21	42	10.3	5.20	0.14	0.0453	
Two to Three	mild	62.8	3.40	54	12.2	5.20	0.03	0.0099	
Three to Four	mild	75.3	3.36	45	11.5	5.20	0.07	0.0192	
Four to Five	marked	89.3	4.18	47	5.3	5.20	0.28	0.0345	
	faint				4.9	5.20	0.19	0.0184	
One to Two	marked	52.9	3.20	61	14.9	5.20	0.20	0.0781	
Two to Three	faint	62.8	3.43	55	9.8	5.20	0.13	0.0322	
Three to Four	mild	75.3	3.82	51	19.6	5.20	0.04	0.0255	

Four to Five	marked	89.3	1.60	18	14.1	5.20	0.33	0.2146	
	faint				4.7	5.20	0.21	0.0168	
One to Two	faint	52.9	3.07	58	11.6	5.20	0.03	0.0047	
Two to Three	marked	62.8	3.11	50	9.6	5.20	0.32	0.1039	
Three to Four	marked	75.3	4.22	56	19.0	5.20	0.09	0.0538	
Four to Five	mild	89.3	2.21	25	16.4	5.20	0.23	0.1220	
	faint				9.7	5.20	0.11	0.0351	
One to Two	marked	52.9	2.33	44	8.7	6.20	0.13	0.0376	
Two to Three	mild	62.8	3.52	56	16.0	6.20	0.06	0.0367	
Three to Four	mild	75.3	4.10	54	14.1	6.20	0.08	0.0414	
Four to Five	marked	89.3	3.52	39	16.0	6.20	0.07	0.0437	
	mild				14.9	6.20	0.12	0.0602	
One to Two	mild	52.9	3.12	59	3.2	4.90	0.48	0.0103	5
	marked				8.2	4.90	0.42	0.0994	
Two to Three	mild	62.8	3.86	61	19.4	4.90	0.13	0.0828	
Three to Four	mild	75.3	4.33	58	18.3	4.90	0.04	0.0268	
	marked				5.2	4.90	0.20	0.0109	
	mild				0.5	4.90	1.49	0.0021	
	marked				1.6	4.90	0.51	0.0064	
One to Two	faint	62.8	3.36	54	3.3	5.20	0.34	0.0167	5
Two to Three	faint	75.3	4.83	64	8.3	5.20	0.09	0.0151	9
Three to Four	mild	89.3	2.59	29	10.5	5.20	0.08	0.0234	10
	mild				7.7	5.20	0.16	0.0341	8
One to Two	faint	62.8	4.39	70	7.2	4.70	0.07	0.0085	
Two to Three	mild	75.3	3.71	49	8.3	4.70	0.08	0.0154	
Three to Four	mild	75.3	5.16	68	11.9	4.70	0.29	0.0873	
	marked	89.3			8.3	4.70	0.31	0.0667	15
One to Two	faint	62.8	4.24	68	13.9	5.40	0.33	0.1797	
Two to Three	swale	75.3	4.54	60	3.4	5.40	0.27	0.0207	
	mild				7.6	5.40	0.11	0.0298	

Observed
PK inter-
LEH
79

43
55
33
